# Supplementary material for: Low-Dose Sorafenib Promotes Cancer Stem Cell Expansion and Accelerated Tumor Progression in Soft Tissue Sarcomas
Source: Int J Mol Sci. 2024 Mar 15;25(6):3351. doi: 10.3390/ijms25063351 (PMC10969893; doi:10.3390/ijms25063351)
Supplement: Supplementary file 1 [file ijms-25-03351-s001.zip › Cruz et al. Supplemental Figure.pdf]

Supplemental figure

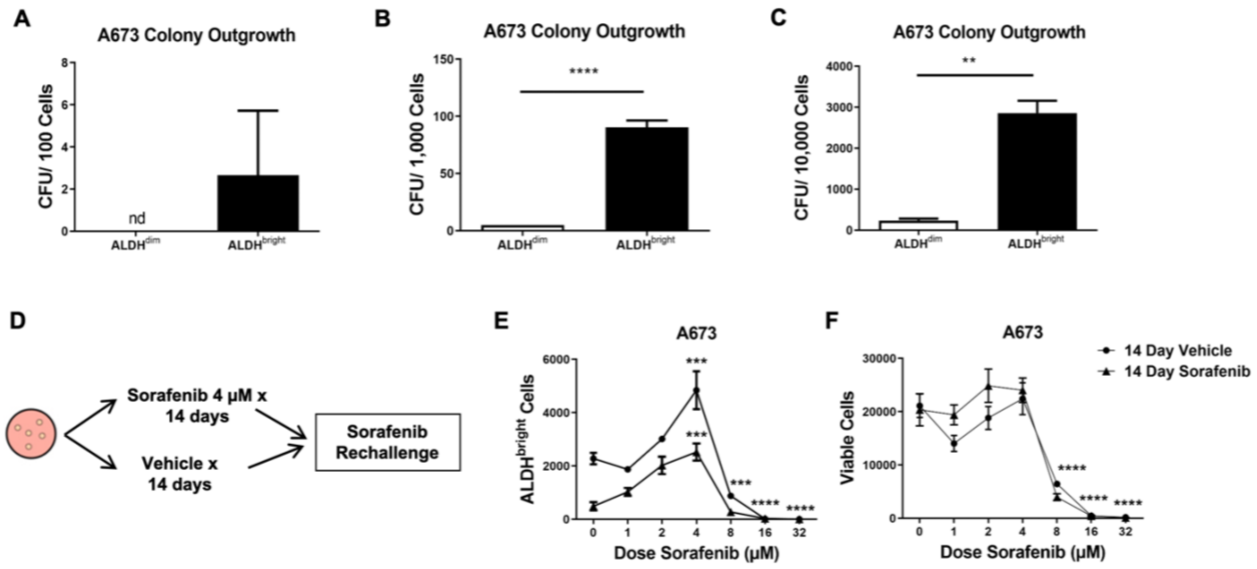

<u>Supplemental Figure S1. $\text{ALDH}^{\text{bright}}$ A673 cells show stem-like behavior in vitro and in vivo.</u> (A – C) A673 Ewing's sarcoma cells were sorted into $\text{ALDH}^{\text{bright}}$ and ALDH^{dim} populations and plated in soft agar plates at indicated concentrations. Colony forming units were counted and scored after 6 days. (D) Schema depicting a 14-day in vitro experiment. A673 Ewing's sarcoma cell line was plated prior to rechallenge at varying doses. (E – F) Treated and untreated cells were then assessed for $\text{ALDH}^{\text{bright}}$ and viable cells were measured by flow cytometry using the ALDEFLUOR™ assay and 7-AAD, respectively. All experiments were performed in triplicate. *$P < 0.05$, **$P < 0.01$, ***$P < 0.001$, ****$P \leq 0.0001$ via one-way ANOVA with Tukey's post-test.
