# Supplementary material for: Low-Dose Sorafenib Promotes Cancer Stem Cell Expansion and Accelerated Tumor Progression in Soft Tissue Sarcomas
Source: Int J Mol Sci. 2024 Mar 15;25(6):3351. doi: 10.3390/ijms25063351 (PMC10969893; doi:10.3390/ijms25063351)
Supplement: Supplementary file 1 [file ijms-25-03351-s001.zip › Supplemental Methods.docx]

**Supplemental Methods**

*Reagents*

Sorafenib p-Tosylate salt and pazopanib free base were purchased from LC Laboratories (Woburn, MA). For in vitro experiments, the lyophilized compounds were dissolved in 100% DMSO and then diluted to final concentration of 0.2% DMSO. Stock solutions were replenished every 4–6 weeks per manufacturer’s recommendations. Further information on key reagents and resources is listed in **Table S1**.

**Table S1. Key Resources**

| REAGENT or RESOURCE | SOURCE | IDENTIFIER |
| --- | --- | --- |
| Antibodies | | |
| PE-Cy7 anti-human CD24 (clone ML5) | BioLegend | Cat #: 311119 |
| Pacific Blue anti-human CD44 (clone IM7) | BioLegend | Cat #: 103019 |
| ALDEFLUOR Kit | STEMCELL Technologies | Cat #: 01700 |
| CD133 (clone AC133) | Miltenyi Biotec | Cat #: 130-113-670 |
| Chemicals, peptides, and recombinant proteins | | |
| Sorafenib, p-Toluenesulfonate Salt, >99% | LC Laboratories | Cat #: S-8502, Lot BSF-104 |
| Pazopanib, Free Base, >99% | LC Laboratories | Cat #: P-6706 |
| Hyclone Dulbecco’s Modified Eagle Medium (DMEM) | VMBMS | Cat #: HY010 |
| Fetal Bovine Serum (FBS) | Gemini | Cat #: 100-106 |
| 7-Amino-Actinomycin D (7-AAD) | BD Biosciences | Cat #: 559925 |
| Dimethyl Sulfoxide (DMSO) | SIGMA Life Science | Cat #: D2650 |
| Penicillin Streptomycin (P.S.) | Corning Cellgro | Cat #: 30-001-Cl |
| Critical commercial assays | | |
| RNeasy Mini Kit | Qiagen | Cat #: 74104 |
| High-Capacity cDNA Reverse Transcription Kit | Applied Biosystems | Cat #: 43-688-14 |
| Experimental models: Cell lines | | |
| A673 | American Type Culture Collection | Cat #: CRL-1598 |
| SK-LMS | American Type Culture Collection | Cat #: HTB-88 |
| SW- 982 | American Type Culture Collection | Cat #: HTB-93 |
| PANC-1 | American Type Culture Collection | Cat #: CRL-1469 |
| MDA-MB-231 | American Type Culture Collection | Cat #: HTB-26 |
| ACHN | American Type Culture Collection | Cat #: CRL-1611 |
| Experimental models: Organisms/strains | | |
| Female NOD.Cg-*Prkdc*^scid^ Il2*rg*^tm1Wjl^/SzJ (NSG) Mice | The Jackson Laboratory | Cat #: 005557 |
| Software and algorithms | | |
| GraphPad Prism | https://www.graphpad.com/ | Version 9.4.1 |
| FlowJo | https://www.flowjo.com/ | Version 10.8.1 |
| Other | | |
| RT² SYBR Green qPCR Mastermix | Qiagen | Cat #: 330502 |
| StepOnePlus™ Real-Time PCR system | Applied Biosystems | Cat #: 4376600 |

*Retrospective analysis of clinical trial*

The results of a phase I trial of neoadjuvant radiotherapy plus sorafenib for patients with locally advanced STS of the extremity and body wall were reported previously (Clinical Trial Information: NCT#00805727)^25^. Eight patients were enrolled (five females, median age 44 years, two high-grade pleomorphic, two myxoid/round cell liposarcoma, four other) with a median tumor size of 16 cm (range 8–29). Clinical outcomes were reanalyzed with respect to ALDH staining on archived tumor samples. A contemporaneous cohort of STS patients treated with standard of care preoperative radiotherapy and surgery were analyzed retrospectively with respect to clinical outcomes and ALDH staining under IRB approval for a retrospective analysis. See **Supplemental Protocol** for further details regarding the clinical trial.
