# Supplementary material for: Low-Dose Sorafenib Promotes Cancer Stem Cell Expansion and Accelerated Tumor Progression in Soft Tissue Sarcomas
Source: Int J Mol Sci. 2024 Mar 15;25(6):3351. doi: 10.3390/ijms25063351 (PMC10969893; doi:10.3390/ijms25063351)
Supplement: Supplementary file 1 [file ijms-25-03351-s001.zip › Supplemental Protocol - Sorafenib Trial Protocol (UCD#216).pdf]

Phase I/II Trial of Neoadjuvant Conformal Radiotherapy plus Sorafenib for Patients with Soft Tissue Sarcoma of the Extremity

PRINCIPAL INVESTIGATOR:

Robert J. Canter, MD
Division of Surgical Oncology
UC Davis Cancer Center
4501 X Street, Suite 3010
Sacramento, CA 95817
[redacted]

CO-INVESTIGATORS:

Robert M. Tamurian, MD, FAAOS
Assistant Professor of Orthopaedic Surgery
Chief of Orthopaedic Oncology
University of California at Davis
4860 Y Street, Suite 3800
Sacramento, CA 95817

Abimbola Olusanya, NP
Nurse Practitioner
Division of Surgical Oncology
U C Davis Medical Center
4501 X Street, Suite 3010
Sacramento, CA 95817

Scott Christensen, MD
Hematology-Oncology
UC Davis Cancer Center
4501 X Street
Sacramento, CA 95817
[redacted]

Dariusz Borys, MD
Pathology and Laboratory Medicine
UC Davis Health System
PATH Building, 4400 V Street
Sacramento, CA 95817
[redacted]

Philip Mack, PhD
Molecular Biology and Genetics
UC Davis School of Medicine
Sacramento, CA 95817

Wayne Monsky, MD, PhD
Department of Radiology
Director, Imaging Response Assessment
UC Davis Medical Center
2315 Stockton Boulevard
Sacramento, CA 95817
[redacted]

Allen Chen, MD
Radiation Oncology
UC Davis Cancer Center
4501 X Street, Suite G126
Sacramento, CA 95817
[redacted]

Robert O'Donnell, MD
Hematology-Oncology
UC Davis Cancer Center
4501 X Street
Sacramento, CA 95817
[redacted]

I-Yeh Gong, MD
Hematology-Oncology
UC Davis Cancer Center
4501 X Street
Sacramento, CA 95817
[redacted]

Michael Buonocore, PhD
Department of Radiology
Imaging Center, 1215
Sacramento, CA 95817
[redacted]

<u>STATISTICIAN:</u>

Xiaowen Yang, PhD
Division of Biostatistics
Med Sci 1-C, One Shields Avenue
Davis, CA 95616
[redacted]

TABLE OF CONTENTS

Title Page 1-2
Table of Contents 3-5
Synopsis 6-7
1.0 Introduction
1.1 Soft Tissue Sarcoma……8
1.2 Surgery with External Beam Radiation Therapy……8-9
1.3 Anti-Angiogenic Targeted Therapy……9
1.4 Anti-Angiogenic Agents with Radiation Therapy……9-10
1.5 Treatment-Induced Pathologic Necrosis……10
1.6 Sorafenib……10-11
1.7 Dynamic Contrast-Enhanced Magnetic Resonance Imaging……11
1.8 Molecular Correlative Studies……11-12
2.0 Study Design……12-13
3.0 Objectives
3.1 Primary……13
3.2 Secondary……13-14
4.0 Accrual Goal 14
5.0 Schema 14-15
6.0 Patient Eligibility
6.1 Inclusion Criteria 15-16
6.2 Exclusion Criteria 16-17
7.0 Treatment Plan
7.1 Sorafenib Dose Levels and Definition of Dose-Limiting Toxicity……17-18
7.2 Sorafenib Dose Modification
7.2.1 General Considerations……18-19
7.2.2 Dose Modification for Sorafenib-Associated Hand-Foot Skin Reaction……20
7.2.3 Dose Modification for Sorafenib-Associated Hypertension……21
7.2.4 Dose Modification for Other Sorafenib-Associated Toxicity……21-23
7.3 Sorafenib Interaction with Other Medications……23-24

7.4 Radiation…………………………………………………………….....24
7.5 Surgery…………………………………………………………………..24-25
7.6 Pathologic Evaluation……………………………………………………25
7.7 Duration of Therapy……………………………………………………..25-26
8.0 Agent Formulation and Procurement
8.1 Sorafenib………………………………………………………………26-27
9.0 Correlative/Special Studies
9.1 Dynamic Contrast-Enhanced MRI…………………………………………27
9.2 Serum and Tissue Biomarkers……………………………………………27-29
9.3 Function/Quality of Life Measurements……………………………………29
10.0 Study Calendar/Schedule of Events/Study Procedures
10.1 Study Calendar…………………………………………………………..29
10.2 Subject Registration……………………………………………………29 – 30
10.3 Baseline Screening………………………………………………………..30
10.4 Study Visits
10.4.1 Toxicities and Adverse Events……………………………..……30
10.4.2 Assessment of Wound Complications……………………………30
10.4.3 Follow-up…………………………………………………………31
10.4.4 Schedule of Events………………………………………..31-32
10.5 Specimen Submission for Molecular Correlative Studies………................32-33
11.0 Measurement of Effect
11.1 Pathologic Response……………………………………………………33
11.2 Radiographic and Clinical Response………………………………… 33 – 34
11.3 Response Criteria
11.3.1 Evaluation of Target Lesion ……………………………………34
11.3.2 Evaluation of Best Overall Response……………………………..34
11.4 Time-to-Event Measures…………………………………………….......35
12.0 Ethical and Regulatory Requirements
12.1 Protocol Review…………………………………………………………35
12.2 Informed Consent……………………………………………………….35
12.3 Protocol Changes or Amendments……………………………………35 – 36

12.4 Maintenance of Records……………………………………………………36
12.5 IRB Reporting of Unanticipated Problems and Adverse Events……………36
12.6 MedWatch Reporting……………………………………………………….36
12.7 Inclusion of Women, Minorities, and Children
12.7.1 Inclusion of Women and Minorities…………………………….…37
12.7.2 Inclusion of Children……………………………………….......37
13.0 Statistical Considerations
13.1 Primary Objective
13.1.1 Phase I……………………………………………………..37-38
13.1.2 Phase II……………………………………………………..38
13.2 Secondary Objectives……………………………………………………38-39
14.0 Appendices
14.1.1 ECOG Performance Status…………………………………………………39
14.1.2 IRAT DCE-MRI Protocol……..……………………………………39 – 50
14.1.3 Toronto Extremity Salvage Score…………………………………………..50
14.1.4 SF-36 Health Survey……………………………………………………..50
15.0 References……………………………………………………………50 – 54

SYNOPSIS

**Title**	Phase I/II Trial of Neoadjuvant Conformal Radiotherapy plus Sorafenib for Patients with Soft Tissue Sarcoma of the Extremity
**Sponsor**	University of California, Davis Medical Center, Division of Surgical Oncology
**Phase**	I/II
**Tumor site**	Soft Tissue Sarcoma of the Extremity and Body Wall<br>• Intermediate or high grade, tumor > 5 cm in maximal dimension (AJCC 7th Edition stage IIb or III)<br>• Low grade, tumor > 8 cm in maximal dimension (AJCCstage Ib)<br>Intervention prior to elective, definitive surgery
**Anticipated accrual**	Phase I: Dose-escalation with traditional 3+3 cohort design, 6 to 12 patients.<br>Phase II: 25 patients (anticipated)
**Intervention**	Patients will receive neoadjuvant sorafenib (investigational agent) in combination with preoperative external beam conformal radiotherapy (50 Gy in 25 fractions) for localized, large soft tissue sarcomas of the extremity prior to resection with curative intent. Sorafenib is an FDA-approved targeted agent for patients with renal cell carcinoma and hepatocellular carcinoma. Preliminary data suggest activity for sorafenib against soft tissue sarcoma in the metastatic setting. Limited data are available regarding the safety and efficacy of sorafenib in combination with radiotherapy.<br><br>The Phase I portion of the trial will seek to establish the safety of sorafenib and radiotherapy in the neoadjuvant setting for soft tissue sarcomas of the extremity.<br><br>The Phase II portion of the trial will aim to determine the pathologic near-complete/complete response rate (≥ 95% tumor necrosis) of this multimodality therapy. Molecular and dynamic contrast-enhanced MRI studies will seek to establish correlative biological and imaging markers of response and/or resistance to this therapy.
**Rationale**	Significant challenges exist in the treatment of patients with soft tissue sarcoma (STS) of the extremity. Major therapeutic goals for all patients include local disease-control, maximization of limb function, and avoidance of therapeutic morbidity. The standard approach for patients with STS has been based on radical resection with wide margins in combination with external beam radiation. Although local control rates in this setting range from 85 to 90%, patients remain at risk for substantial morbidity from this multimodality therapy. Furthermore, despite effective local therapy with surgery and radiation, approximately 50% of patients with high grade STS will die of disease within 5 years of diagnosis.<br><br>Experimental data and experience from other solid tumors suggest a significant synergistic effect when anti-angiogenic targeted therapies, such as sorafenib, are combined with radiotherapy (RT). Overproduction of angiogenic factors in the

	tumor vasculature leads to disordered/poorly regulated tumor perfusion which appears to normalize following delivery of anti-angiogenic agents. This, in turn, allows improved delivery of and susceptibility to cytotoxic agents, which is particularly important with RT since hypoxia is a key mechanism of resistance to this modality. Preliminary clinical data in rectal cancer and malignant brain tumors have demonstrated promising results from the combination of anti-angiogenic agents and RT with respect to downstaging of the primary tumor, local tumor control, and improved progression-free survival. Angiogenic factors are upregulated in patients with STS, and anti-angiogenic agents demonstrate activity against STS in the metastatic setting.<br><br>Consequently, we propose to evaluate preoperative combined modality sorafenib and conformal RT in patients with STS of the extremity in this prospective trial. Key endpoints will be safety and toxicity in the Phase I trial and pathological response in the Phase II trial. Additional endpoints will include molecular and functional imaging correlative studies of biomarkers of response and resistance to treatment. Enrollment into Phase I will be completed prior to the initiation of phase II.<br><br>We hypothesize that this combination will be safe and will increase pathologic necrosis within the primary tumor at the time of surgical resection, translating into reduced volume of resected tissue, improved local disease-control, and ultimately improved disease-free survival. Functional imaging studies (dynamic contrast-enhanced MRI) and molecular examination of pre- and post-treatment tumor tissue and serum will allow correlation of changes in tumor blood flow, hypoxia transcription factors, phosphorylated ERK, and angiogenic markers to the degree of tumor necrosis following treatment with preoperative sorafenib and radiotherapy.

1.0 INTRODUCTION

1.1 Soft Tissue Sarcoma

Soft tissue sarcomas (STS) comprise a diverse group of tumors of mesenchymal differentiation which together account for approximately 10,000 cancer cases per year in the United States.[1] Significant challenges exist in the care of these patients. Competing therapeutic goals include maximization of local and distant-disease control with avoidance of morbidity. Although function/limb-sparing surgery combined with radiotherapy (RT) has successfully replaced amputation in 90% to 95% of patients with local control rates of 76% to 92%,[2] patients remain at risk for substantial morbidity from this multimodality therapy. Moreover, patients with high grade tumors have a risk of distant recurrence and death as high as 50% within 5 years of diagnosis.[3] Furthermore, since the adoption of combined function-sparing surgery with RT for STS over 25 years ago, despite increased knowledge regarding the molecular biology and genetics of STS,[4] few significant advances have been made in the treatment of patients with STS of the extremity.[2, 5] Therefore, novel therapeutic approaches are needed.[4]

1.2 Surgery with External Beam Radiotherapy

For patients with localized STS amenable to resection with curative intent, surgical treatment in combination with RT results in local disease control in 76% to 92% of patients with successful limb preservation, i.e. avoidance of amputation, in 90 to 95% of patients whose tumors are located on the extremity.[2] Although function-sparing surgery combined with RT represents a significant advance for patients with STS, this approach still requires total en bloc excision of the primary tumor with a goal of 2 centimeters of normal tissue around the tumor in all directions. These operations risk significant morbidity for the patient.

An important component of local therapy in STS is adjuvant RT. Adjuvant RT for patients with STS of the extremity has been shown to reduce local recurrence rates by 20 to 25% relative to radical resection alone.[6, 7] The timing of RT administration (preoperative versus postoperative) has been compared in a randomized clinical trial.[8, 9] This trial demonstrated that preoperative RT was associated with a statistically significant increase in perioperative wound complications (35% vs. 17%).[8] Conversely, at a median follow-up of 6.9 years, preoperative RT was associated with a statistically significant decrease in long term complications, such as joint fibrosis, joint stiffness, and extremity edema (23% vs. 36%).[9] Although these data have been interpreted in different ways by different investigators,[2] advocates of preoperative radiotherapy maintain that the data favor the preoperative administration of RT since perioperative wound complications are generally reversible whereas late complications of radiotherapy are generally irreversible. Furthermore, although considered a secondary endpoint of the trial, overall survival was statistically improved in the patients who received preoperative radiotherapy (85% vs. 75% at 3 years, P=0.048).[8] Although these secondary survival data regarding neoadjuvant RT should be viewed with caution, it is reasonable to hypothesize that neoadjuvant RT in combination with novel systemic agents may improve both local and distant disease-control in patients with STS.

This is particularly relevant for patients with high grade primary STS greater than 5 centimeters in maximal dimension (AJCC stage IIb and III). These patients carry a risk of distant recurrence and death that approaches 50% at 5 years.[3, 10, 11] Studies utilizing traditional chemotherapeutic agents, such as anthracyclines and ifosfamide, either alone or in combination with RT, have failed to demonstrate durable benefits either in tumor downstaging to allow a less extensive resection or in patient survival.[12-17] The minority of prospective studies showing survival, local-recurrence, or distant-recurrence benefits to adjuvant chemotherapy are mitigated by numerous studies demonstrating no benefit.[18] Furthermore, a prospective multi-institutional Phase II trial of doxorubicin-ifosfamide chemotherapy interdigitated with RT resulted in 5% treatment-related deaths, 78% grade 4 hematologic toxicity, and 19% grade 4 non-hematologic toxicity.[16] In this trial, the overall survival rate at 3 years was 75%. Given the substantial toxicity, these results do not appear sufficiently favorable compared to historical controls to justify the widespread application of this regimen to patients with STS.

1.3 Anti-angiogenic Targeted Therapy

Angiogenesis and neovascularization is a fundamental component of tumor initiation, promotion, and acquisition of the metastatic phenotype.[19, 20] Overexpression of vascular endothelial growth factor (VEGF), its receptors, and other angiogenic proteins (e.g. fibroblast growth factor) has been observed as a near universal neoplastic phenomenon.[21] Correspondingly, anti-angiogenic agents, such as bevacizumab, have demonstrated statistically significant increases in patient survival in randomized, controlled clinical trials in numerous settings, including colorectal, breast, and non-small cell lung carcinomas.[22-24] Although not typically tested as monotherapy, a fundamental aspect of these positive results for anti-angiogenic agents appears to be the combination with conventional chemotherapy.

Similar to other tumor histologies, STS have been shown to overexpress angiogenic factors in both tumor tissue and serum,[25-29] suggesting that anti-angiogenic therapy may prove effective for STS patients as well. Retrospective studies have demonstrated tumor and serum vascular endothelial growth factor (VEGF) concentration to correlate with grade, stage, and prognosis of STS.[25, 28, 30] In addition, anti-angiogenic therapy with bevacizumab in combination with conventional chemotherapy has shown activity against STS in the metastatic setting.[31] Data such as these support the hypothesis that angiogenesis inhibition with targeted agents will demonstrate activity in the adjuvant/neoadjuvant setting for patients with STS.

1.4 Anti-Angiogenic Agents and RT

There are limited data examining the combination of RT and anti-angiogenic agents, particularly in STS. Pre-clinical data suggest a significant synergistic effect when anti-angiogenic targeted therapies, such as sorafenib, are combined with RT.[32, 33] Overproduction of angiogenic factors in the tumor vasculature leads to disordered/poorly regulated tumor perfusion. Delivery of anti-angiogenic agents appears to "normalize" the tumor micro-environment with subsequent enhanced efficacy of cytotoxic treatments on tumor tissue.[20]

This mechanism is particularly relevant for RT since hypoxia is a crucial mediator of resistance to this therapeutic modality.

Preliminary clinical data in rectal cancer and malignant brain tumors have demonstrated promising results for the combination of anti-angiogenic agents and RT with respect to downstaging of the primary tumor, local control, and improved progression-free survival.[34-36] Although tumors may change little in overall size with targeted agents[37], pathologic analysis frequently demonstrates significant tumor necrosis, and in some cases no viable residual tumor may be present. These findings are significant for STS since complete (100% tumor necrosis) or near-complete (≥ 95% tumor necrosis) pathologic response has been observed, in a retrospective analysis, to correlate with improved local, distant, and overall survival in STS patients treated with neoadjuvant chemoradiotherapy using doxorubicin and ifosfamide.[38]

1.5 Treatment-Induced Pathologic Necrosis

When used as monotherapy, preoperative RT has rarely been observed to lead to significant rates of complete or near-complete pathological response. Two single institution series have reported rates of 80% tumor necrosis in approximately 30% of patients.[39, 40] However, few complete (100% tumor necrosis) or near-complete (≥ 95% tumor necrosis) pathologic responses were observed, and, although limited and retrospective, the data suggest that < 95% tumor necrosis is not associated with significant improvements in clinical endpoints, such as local-disease control, distant-disease control, or overall survival.[38]

1.6 Sorafenib

Sorafenib (BAY 43-9006) is an oral multi-kinase inhibitor with effects on tumor proliferation and tumor angiogenesis. It was initially selected based on inhibitory activity against the serine/threonine kinases Raf-1 and wild-type B-Raf, which are pivotal components of the Ras/Raf/MEK/ERK signaling pathway. Inhibitory activity was subsequently demonstrated against the tyrosine kinases for the VEGF receptor and the platelet-derived growth factor (PDGF) receptor in addition to Flt-3 and c-Kit.[41]

In preclinical studies, sorafenib has demonstrated broad-spectrum anti-tumor activity by inducing complete tumor stasis and inhibition of tumor angiogenesis in a variety of tumor types.[41] In the murine renal adenocarcinoma and VHL mutated xenograft models, sorafenib prevented tumor growth, primarily through the inhibition of tumor-cell-induced angiogenesis and also induced tumor apoptosis and necrosis.[42]

The safety and clinical activity of sorafenib, alone or in combination with chemotherapy, has been examined in a series of phase I studies conducted in patients with solid tumors.[43, 44] In clinical trials establishing the efficacy of sorafenib monotherapy for patients with renal cell carcinoma and hepatocellular carcinoma, fewer than 10% of patients discontinued therapy because of toxicity, and no patients died from toxicity.[45, 46] Consequently, given its favorable toxicity profile, its anti-angiogenic properties, and its

potential synergistic effect with RT (an established treatment modality for STS),[32, 33] sorafenib appears to be a good candidate for further evaluation in a neoadjuvant clinical trial of novel treatment approach for patients with STS.

1.7 Dynamic Contrast-Enhanced Magnetic Resonance Imaging

Variability in specific patient and tumor molecular factors likely influences the response or resistance of individual patients to treatment. Consequently, novel methods for evaluating and monitoring tumor response to therapy are needed beyond that of traditional tumor growth or shrinkage. Progress in this arena will enable novel anti-cancer therapies to be individualized for patients in a rational manner to maximize the benefit-to-risk ratio.

A major challenge facing studies of anti-angiogenic therapies is the detection of meaningful drug response in the early-phase after drug introduction. Treatment with these agents is not commonly associated with significant radiographic tumor shrinkage, and no standard surrogate markers for response to these therapies exist.[37] One potential avenue for monitoring activity is functional imaging modalities that can detect changes in blood flow, vessel wall permeability, or metabolic activity that may occur quickly.[47] Dynamic contrast-enhanced magnetic resonance imaging (DCE-MRI) is one such approach that can evaluate changes in the tumor vasculature. In this technique, MRI is used to track bolus injections of paramagnetic gadolinium chelate contrast reagent as it passes through the tumor tissue.[47-49] Concentration time curves are modeled based on signal intensity data in the region of interest. Quantitative values, such as the volume transendothelial transfer constant (K^{trans}) and the extravascular leakage space (v_e), are then determined.[49] Vascularized tumor volume can then be obtained by summing voxels, and histogram and principal components analysis can be employed to assess regional and chronologic change.[47, 48]

DCE-MRI is being actively evaluated as a noninvasive pharmacodynamic biomarker of effect in early-phase clinical trials of antivascular agents.[50, 51] In addition to detecting vascular changes from antiangiogenic therapy, DCE-MRI may also have potential for predicting therapeutic effect from chemoradiotherapy in soft-tissue sarcomas. Several studies in bone sarcomas have shown that histologic response to neoadjuvant chemotherapy can be detected by changes in DCE-MRI contrast-enhancement parameters, such as the initial slope and initial area under the time-intensity curve.[52-54] These findings suggest that DCE-MRI has the potential to be a non-invasive indicator of early response (low permeability/low Ktrans corresponds to favorable prognosis whereas high permeability/high Ktrans corresponds to unfavorable prognosis). These parameters may eventually prove useful in changing treatment approaches for individual patients, and one could postulate a paradigm of modifying preoperative systemic therapy or pursuing more aggressive surgical margins in patients in whom DCE-MRI suggested a poor response to preoperative therapy. However, the technique of DCE-MRI and its ability to predict response to therapy must first be validated in a prospective clinical trial, prior to designing and testing treatment algorithms based on its results.

1.8 Molecular Correlative Studies

The primary objective of the Phase II portion of this trial, the rate of near-complete (≥ 95% tumor necrosis) pathological response to combined neoadjuvant conformal radiotherapy and sorafenib, aims to provide a clinically meaningful endpoint. However, it is clear that correlative markers that allow for a better understanding of the biologic effects of combined sorafenib and RT are an important component of this trial, and for future trials designed to test novel therapies for patients with STS. Multiplex platforms, such as the Luminex system, provide an accurate and rapid mechanism to simultaneously assess concentrations of multiple proteins in patient plasma. Ongoing research within the Southwest Oncology Group (SWOG) is exploring the potential utility of this technique to improve understanding of the pharmacodynamic effects of angiogenesis-targeted agents on circulating pro-angiogenic factors and to identify patient plasma profiles predictive of response.

At UC Davis, we are investigating the utility of a panel of angiogenesis-related factors (VEGF, OPN, PDGF, bFGF, IL-8, IL-6, among others) using the Luminex platform. Preliminary studies of patient plasma using this high-throughput technique demonstrate a highly significant correlation to measurement of VEGF plasma levels by conventional ELISA methods (r^2=0.9359; p <0.0001). Since single angiogenic markers in isolation appear to be unreliable predictors of STS behavior[25], it is hypothesized that angiogenic profiles may be more predictive of response or resistance to anti-angiogenic therapy than individual factors alone. Furthermore, treatment-induced changes in plasma profiles may help identify mechanisms of resistance to angiogenic blockade.

In addition to its anti-angiogenic properties, sorafenib inhibits tumor cell proliferation through the Raf/MEK/ERK signaling pathway.[33] Since hypoxia has profound effects on radioresistance, presumed to be mediated in part through the HIF-1α transcription factor, and since there is significant cross-talk between these pathways[55, 56], it is hypothesized that changes in the immunohistochemical expression of these proteins will predict response, or conversely resistance, to treatment. Tumor tissue pre- and post-multimodality therapy will be tested for immunohistochemical changes in the expression of:

- hypoxia/angiogenic tumor proteins (HIF-1α, VEGFR 1-3, PDGFR)
- growth factors correlated with hypoxia and resistance to radiation (VEGF, EGF, FGF, PDGF)
- modulators of proliferation and signal transduction (Ki-67, pERK, pAKT, mTOR)

In addition to the molecular studies mentioned above, we also propose to take a systematic approach to specimen collection for future testing of yet-to-be identified biomarkers. Specimen collection will include acquisition of tumor specimens pre- and post-treatment, serial plasma samples, and PBMCs.

2.0 STUDY DESIGN

This is a phase I/II, open label, non-randomized study of neoadjuvant conformal radiotherapy plus sorafenib for patients with extremity STS high grade greater than 5 cm in

maximal dimension or low grade greater than 8 cm in maximal dimension.

3.0 OBJECTIVES

3.1 Primary Objectives

- Phase I—to determine the maximal tolerated dose (MTD) of sorafenib when combined with conformal external beam radiation prior to resection with curative intent of > 5 cm high grade or > 8 cm low grade soft tissue sarcomas of the extremity.

- Phase II—to determine the rate of near-complete (≥ 95% tumor necrosis) pathological response to combined neoadjuvant conformal radiotherapy and sorafenib at MTD from Phase I following resection with curative intent of > 5 cm high grade or > 8 cm low grade soft tissue sarcomas of the extremity.

3.2 Secondary Objectives

- Phase I and II

  1. To describe the toxicities associated with neoadjuvant conformal RT in combination with sorafenib.
  2. To determine the rate of R0 (negative resection margin), R1 (microscopically positive resection margin), and R2 (macroscopically resection margin) following neoadjuvant conformal radiotherapy in combination with sorafenib.
  3. To determine the radiographic response rate to combined neoadjuvant conformal radiotherapy and sorafenib as defined by conventional RECIST criteria (**see section 11.3 for definition of RECIST criteria**).
  4. To obtain preliminary data regarding local disease control, distant disease control, progression-free survival, and overall survival with this novel therapeutic regimen.
  5. To assess functional and quality of life outcomes before, during, and after therapy using the established metrics including the Toronto Extremity Salvage Score (appendix 14.1.3) and the SF36 Health Survey (appendix 14.1.4)

- Phase II only
  1. To initiate analyses of molecular markers of tumor response assessment comparing (1) a microarray of a panel of serum angiogenic markers pre- and post-treatment; and (2) immunohistochemistry of tumor tissue pre- and post-treatment for expression of angiogenic proteins (VEGF, VEGFR1-3), growth factors correlated with hypoxia and resistance to radiation (EGF, FGF, HIF-1α), and modulators of proliferation and signal transduction (Ki-67, pERK).
  2. To analyze changes in tumor blood flow, blood volume, capillary permeability, and cell viability pre- and post-treatment with combined sorafenib/RT using dynamic contrast-enhanced MRI. The primary

endpoints of these studies will be the transfer constant (Ktrans) and the initial area under the gadolinium concentration time curve (IAUGC, mMGd◦min—**see section 9.1 for DCE-MRI imaging protocol)**.

4.0 ACCRUAL GOAL

- Phase I— Dose-escalation schedule comprising 6 to 12 patients (see schema). This sample size is based on a traditional 3+3 cohort design with escalating doses of sorafenib in combination with 50 Gy of conformal radiotherapy delivered in 25 fractions (200 cGy per fraction). Based on preclinical data regarding the radiobiology of sorafenib,[33, 36] sorafenib will be initiated at a dose of 200 mg twice daily, followed by 200 mg Q AM/400 mg Q PM for the 2nd cohort, followed by 400 mg bid for the 3rd cohort. Since 400 mg bid is the well established MTD for sorafenib monotherapy in patients with renal cell carcinoma and hepatocellular carcinoma, the dose will not be escalated above this level even if DLT is not observed. Dose level escalation will be determined based on DLTs observed from initiation of sorafenib/RT until time of surgery.

- Phase II— anticipated number of 25 patients will be enrolled depending on phase I result. Anticipating an improvement in near-complete pathological response (≥ 95% tumor necrosis) from 0% with RT alone to 33% with combined sorafenib and RT, we will have an 80% power to detect this difference if we accrue 25 patients, assuming a type I error rate of 5%.

Total target accrual is 30-35 patients depending on results of dose-escalation schedule from Phase I of trial.

5.0 SCHEMA

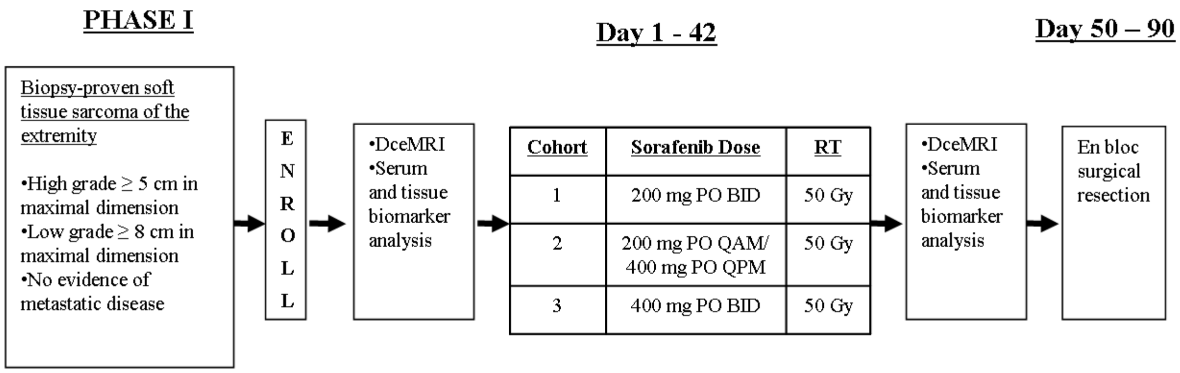

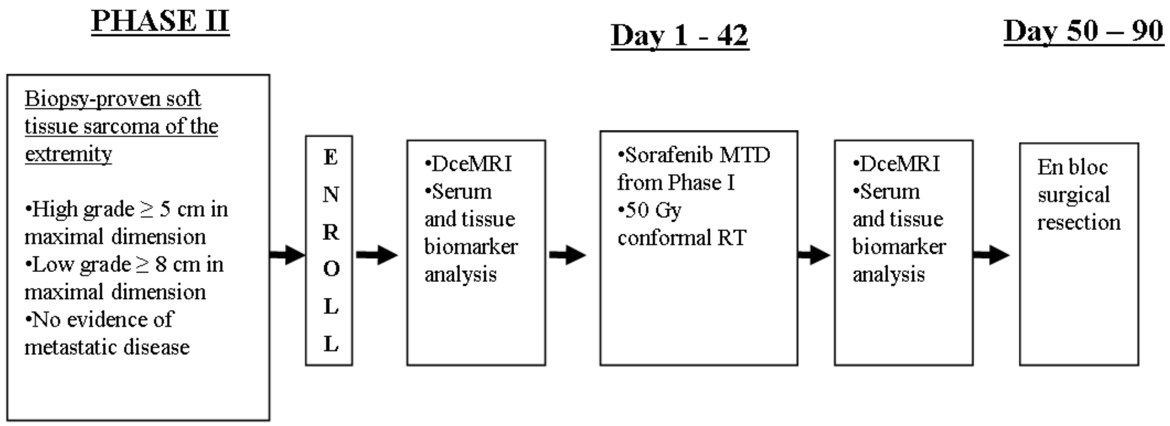

6.0 PATIENT ELIGIBILITY

6.1 Inclusion Criteria

1. Histologically confirmed soft-tissue sarcoma located on the extremity.
    a. Intermediate or High grade (NCI grade 2 or 3/3-tier system), > 5 cm in maximal dimension.
    b. Low grade (NCI grade 1/3-tier system), > 8 cm in maximal dimension.
    c. No evidence of regional or distant metastatic disease.
2. Patient must be 18 years of age or older.
3. Patient must have an ECOG performance status of ≤ 2.
4. Patient must have adequate bone marrow, liver, and renal function as assessed by the following:
    a. Hemoglobin ≥ 8.5 g/dl
    b. Absolute neutrophil count (ANC) ≥ 1,500/mm^3
    c. Platelets ≥ 100,000/mm^3.
    d. Total bilirubin ≤ 1.5 mg/dL. NOTE: Patients with elevated bilirubin secondary to Gilbert's syndrome are eligible to participate in the study.
    e. AST and ALT ≤ 2.5 times the institution upper limit of normal (ULN).
    f. Creatinine ≤ 1.5 times ULN.
5. Females of childbearing potential must have a negative serum or urine pregnancy test within 7 days prior to the start of treatment . NOTE: Postmenopausal women must be amenorrheic for at least 12 months to be deemed not of reproductive potential.
6. Women of childbearing potential and men must agree to use adequate contraception (barrier method of birth control) prior to study entry and for

the duration of study participation. Men should use adequate birth control for at least three months after the last administration of sorafenib.

7. Ability to understand and the willingness to sign a written informed consent. A signed informed consent must be obtained prior to any study specific procedures.
8. INR < 1.5 or a PT/PTT within normal limits. Patients receiving anti-coagulation treatment with an agent such as warfarin or heparin **may** be allowed to participate. For patients on warfarin, the INR should be measured prior to initiation of sorafenib and monitored at least weekly, or as defined by the local standard of care, until INR is stable.
9. If patient is a cancer survivor, ALL of the following criteria must apply:
   a. Patient has undergone potentially curative therapy for all prior malignancies.
   b. No evidence of any prior malignancies for at least 5 years with no evidence of recurrence (except for effectively treated basal cell or squamous cell carcinoma of the skin, carcinoma-in-situ of the cervix treated by surgery alone, or carcinoma-in-situ of the breast treated by surgery alone).
   c. Patient is deemed by their treating physician to be at low risk for recurrence from prior malignancies.

6.2 Exclusion Criteria

A patient will NOT be eligible for inclusion in this study if ANY of the following criteria apply:

1. Patient is receiving additional cancer-directed therapy at time of entry into trial.
2. Any prior systemic therapy for sarcoma
3. Patient has received or is receiving preoperative investigational treatment.
4. Patient has congestive heart failure > class II NYHA. Patients must not have unstable angina (anginal symptoms at rest) or new onset angina (began within the last 3 months) or myocardial infarction within the past 6 months.
5. Patient has history of cardiac ventricular arrhythmia requiring ongoing anti-arrhythmic therapy.
6. Patient with a histologic diagnosis of alveolar rhabdomyosarcoma, embryonal rhabdomyosarcoma, or Ewing's sarcoma.
7. Patient has uncontrolled hypertension, defined as systolic blood pressure > 150 mmHg or diastolic pressure > 90 mmHg, despite optimal medical management.
8. Patient has known human immunodeficiency virus (HIV) infection or chronic Hepatitis B or C.
9. Patient has active clinically serious infection > CTCAE Grade 2.
10. Patient has thrombotic or embolic events such as a cerebrovascular accident including transient ischemic attacks within the past 6 months.

11. Patient has history of pulmonary hemorrhage/bleeding event ≥ CTCAE Grade 2 within 4 weeks of first dose of study drug.
12. Patient has history of any other hemorrhage/bleeding event ≥CTCAE Grade 3 within 4 weeks of first dose of study drug.
13. Patient has history of clinical or laboratory evidence of bleeding diathesis or coagulopathy.
14. Patient has history of major surgery or significant traumatic injury within 4 weeks of first study drug.
15. Patient has concomitant use of St. John's Wort or rifampin (rifampicin).
16. Previous use or current use of Sorafenib.
17. Patient has known or suspected allergy to sorafenib or any agent given in the course of this trial.
18. Patient has any condition that impairs his or her ability to swallow whole pills.
19. Patient has active malabsorption problem not controlled with medical therapy.
20. Indwelling metal that would preclude patient from undergoing DCE-MRI.

7.0 TREATMENT PLAN

7.1 Sorafenib Dose Levels and Definition of Dose-Limiting Toxicity

Evaluation for loco-regional toxicity from therapy will occur daily (excluding weekends and holidays) while patient is receiving RT (**see section 10.1 for study calendar**) and then biweekly during the rest period following completion of RT prior to surgical resection. Evaluation for generalized/systemic toxicity (e.g. hypertension, myelosuppression, etc.) will occur weekly (**see section 10.1 for study calendar**) during the treatment phase and then biweekly during the rest phase of the study. Patients will maintain a pill diary so that a record of self-administered medications will be available.

**Dose Level**	**Sorafenib**	**Radiation**
1	200 mg twice daily	200 cGy x 25 fractions
2	200 mg q am 400mg q pm	200cGy x 25 fractions
2	400 mg twice daily	200 cGy x 25 fractions

A traditional dose-escalation design will be used to determine the MTD of sorafenib combined with radiotherapy. The MTD is defined as the dose that produces dose limiting toxicity (DLT) in 33% of patients. Three patients will be enrolled per dose level, with escalation to the next dose level if no dose-limiting toxicity (DLT) is observed. If one DLT is observed, the dose level will be expanded to a total of 6 patients, and escalation will occur if no more than one DLT is observed among the 6 patients. If 2 or more out of 3 patients, or 3

or more out of 6 patients experience DLT, the study will be terminated or dose reduced to the previous dose level. Dose level escalation will be determined based on DLTs observed from start of treatment until date of surgery, but DLTs will be monitored in the postoperative period, and dose de-escalation may occur if excess late DLTs are observed.

DLT is defined as: grade 4 anemia, grade 4 neutropenia lasting > 7 days, grade 3-4 thrombocytopenia with clinically significant bleeding or grade 4 thrombocytopenia lasting > 5 days, and grade 3-4 non hematologic toxicity (except: nausea/vomiting that responds to anti-emetic therapy; grade 3 neutropenic fever lasting ≤5 days; grade 3 encephalopathy lasting ≤2 days; grade 3-4 hypokalemia, hypophosphatemia, hypomagnesemia, hypocalcemia unless requiring hospitalization; grade 3 diarrhea controlled with medication within 2 days; and grade 3 grade hypertension that is adequately controlled with medication (BP <150/100)).

Treatment Stop Rule

Furthermore, a pause in study accrual will be implemented in the phase II portion of the trial after 10 patients have completed concurrent sorafenib plus conformal radiotherapy followed by surgical resection. Safety assessment will be made 6 weeks following resection of the 10th patient (9 – 13 weeks following completion of concurrent sorafenib and RT) to ensure that excess DLTs are not observed. The study will be ***terminated for safety*** (not lack of efficacy) if 4 of these 10 patients experience grade 3 adverse events or greater since this would represent an increase from the baseline rate of serious AEs expected from the standard combination therapy of preoperative radiation and surgical resection.[8] This pause in accrual will allow us to review all safety data collected to date. If DLTs in excess of our stopping rule are not observed, then the phase II trial will continue to the targeted accrual of 25 patients.

7.2 Sorafenib Dose Modification

7.2.1 General Considerations. According to the following dose level table, sorafenib dosing will be modified for adverse events that are at least in-part *attributable* to sorafenib according to the following sections.

**Dose Level**	**Sorafenib**
0*	200 mg daily
1	200 mg twice daily
2	200mg am 400mg pm
3	400 mg twice daily

*Patients will be removed from protocol treatment if dose reduction below this level is indicated.

7.2.2 Dose Modifications for Sorafenib for Hand-Foot Skin Reaction

Grade	Occurrence	Suggested Dose Modification
Grade 1<br>Numbness, dysesthesia, paresthesia, tingling, painless swelling, erythema or discomfort of the hands or feet which does not disrupt the patient's normal activities	Any	Promptly institute supportive measures such as topical therapy for symptomatic relief and continue sorafenib treatment
Grade 2<br>Painful erythema and swelling of the hands or feet and/or discomfort affecting the patient's normal activities	First	Promptly institute supportive measures such as topical therapy for symptomatic relief and consider a decrease of sorafenib by one dose level for a minimum of 7 days and up to 28 days<br>• If toxicity resolves to grade 0–1 after dose reduction, increase sorafenib back to previous dose level<br>• If toxicity does not resolve to grade 0-1 despite dose reduction, interrupt sorafenib treatment for a minimum of 7 days and until toxicity has resolved to grade 0–1.<br>▪ When resuming treatment after dose interruption, resume sorafenib at a one dose level reduction<br>▪ If toxicity is maintained at grade 0–1 at reduced dose for a minimum of 7 days and up to 28 days, increase back sorafenib to previous dose level
	Second or Third occurrence	As for first occurrence. Upon resuming sorafenib, decrease dose by one dose level .<br>Decision whether to dose re-escalate should be based on clinical judgment and patient preference.
	Fourth occurrence	Decision whether to discontinue sorafenibt should be made based on clinical judgment and patient preference
Grade 3<br>Moist desquamation, ulceration, blistering or severe pain of the hands or feet, or severe discomfort that causes the patient to be unable to work or perform activities of daily living	First occurrence	Institute supportive measures such as topical therapy for symptomatic relief and interrupt sorafenib treatment for a minimum of 7 days and until toxicity has resolved to grade 0–1<br>• When resuming treatment after dose interruption, decrease sorafenib by one dose level<br>• If toxicity is maintained at grade 0–1 at reduced dose for a minimum of 7 days and up to 28 days, increase by one dose level
	Second occurrence	As for first occurrence. Upon resuming sorafenib treatment, decrease dose by one dose level.<br>Decision whether to dose re-escalate should be made based on clinical judgment and patient preference.
	Third occurrence	Decision whether to discontinue sorafenib treatment should be made based on clinical judgement and patient preference.

7.2.3 Dose Modifications for Sorafenib-Associated Hypertension

Hypertension is a known and potentially serious adverse event associated with sorafenib treatment. Patients will have their blood pressure monitored and recorded weekly during the first 6 weeks of therapy

Grade (CTCAE v3.0)	Antihypertensive Therapy	Blood Pressure Monitoring	Sorafenib Dose
Grade 1	None	Routine	No change
Grade 2 (asymptomatic)	Initiate monotherapy (suggest dihydropyridine calcium-channel blocker)	Increase frequency and monitor (by health professional) every 2 days until stabilized	No change
Grade 2 (symptomatic/ persistent)<br>**OR**<br>Diastolic BP > 110 mm Hg<br>**OR**<br>Grade 3	Add agent(s):<br>Ca^{++} channel blocker (if not already used),<br>K+ channel opener (angiotensin blockers),<br>beta-blocker,<br>thiazide diuretic	Increase frequency and monitor (by health professional) every 2 days until stabilized; continue qod monitoring to stabilization after dosing restarted.	Hold* sorafenib until symptoms resolve and diastolic BP < 100 mm/Hg.<br><br>Resume treatment at 1 dose level lower
Grade 4			Off protocol therapy
* Patients requiring a delay of > 21 days should go off protocol therapy			
**CTCAE v3.0 definitions**<br>Grade 1: asymptomatic, transient (<24 hrs) increase by > 20 mmHg (diastolic) or > 150/100 if previously WNL; intervention not indicated<br>Grade 2: recurrent or persistent (>24 hrs) or symptomatic increase by >20 mmHg (diastolic) or to >150/100 if previously WNL; monotherapy may be indicated.<br>Grade 3: requiring more than one drug or more intensive therapy than previously<br>Grade 4: life threatening (*e.g.*, hypertensive crisis)			

7.2.4 Dose Modifications for Other Sorafenib-Associated Toxicity

Toxicity	Grade 1	Grade 2	Grade 3*	Grade 4*
**Non-hematologic**	Continue at the same dose level.	Continue at the same dose level.	Withhold dose until toxicity is grade ≤1, then resume treatment at the same dose level. If patient experiences	Withhold dose until toxicity is grade ≤1, then reduce one dose level and resume treatment, or

			a second grade 3 toxicity, withhold dose until toxicity is grade ≤1, then reduce one dose level and resume treatment.	discontinue at the discretion of the principal investigator.
**Hematologic**	Continue at the same dose level.	Continue at the same dose level.	Withhold dose until toxicity is grade ≤2, then resume treatment at the same dose level. If patient experiences a second grade 3 toxicity, withhold dose until toxicity is grade ≤2, then reduce dose to 400 mg p.o. qd and resume treatment.	Withhold dose until toxicity is grade ≤2, then reduce dose to 400 mg p.o. qd and resume treatment, or discontinue at the discretion of the principal investigator after discussion with study supporter. .

*Patients who develop grade 3 fever/chills, grade 3 elevation of hepatic transaminases with ALT and AST <10X ULN, grade 3 hyperlipasemia or hyperamylasemia without clinical or other evidence of pancreatitis, grade 3 leukopenia, or grade 3/grade 4 lymphopenia may continue study treatment without interruption at the discretion of the investigator.

Because certain grade 3-4 adverse events may occur with radiotherapy, the above table should be modified for these adverse events as defined below (unless the adverse events are definitely attributable only to sorafenib, in which case refer to above table). Attribution to sorafenib at least in-part should be present in order to justify sorafenib dose-reduction:

- Neutropenia – Grade 3 or Grade 4 lasting ≤7 days: no action. Grade 4 lasting >7 days: refer to table above
- Neutropenic Fever – Grade 3 lasting ≤5 days: no action. Grade 3 lasting >5 days or Grade 4: refer to table above
- Leukopenia – No dose reduction
- Thombocytopenia -- Grade 3: no action. Grade 4: refer to table above. Grade 3-4 with clinically significant bleeding: treat as Grade 4
- Anemia – Grade 3: no action. Grade 4: refer to table above
- Nausea/Vomiting – Per above table, only if persistent Grade 3-4 despite maximal anti-emetic adjustment
- Fatigue – Grade 3: no action. Grade 4: refer to table above.
- Diarrhea – Diarrhea should be initially managed with anti-diarrhea agents including loperamide or Lomotil. If persistent Grade 3- 4 toxicity, refer to table above.

- Hypophosphatemia – Grade 3: replacement therapy. Grade 4: refer to above table only if refractory to replacement therapy

7.3 Sorafenib Interaction with Other Medications

Drug-Drug Interactions: Caution is recommended when administering sorafenib together with compounds that are metabolized/ eliminated predominantly by the UGT1A1 pathway (e.g. irinotecan)

- **CYP3A4 inducers:** There is no clinical information on the effect of CYP3A4 inducers on the pharmacokinetics of sorafenib. Inducers of CYP3A4 activity (e.g. rifampicin, Hypericum perforatum also known as St. John's wort, phenytoin, carbamazepine, phenobarbital, and dexamethasone) may increase metabolism of sorafenib and thus decrease sorafenib plasma concentrations.

- **CYP3A4 inhibitors:** Ketoconazole, a potent inhibitor of CYP3A4, administered once daily for 7 days to healthy male volunteers did not alter the mean AUC of a single 50 mg dose of sorafenib. Therefore, clinical pharmacokinetic interactions of sorafenib with CYP3A4 inhibitors are unlikely.

- **CYP2C9 substrates:** The possible effect of sorafenib on the metabolism of the CYP2C9 substrate warfarin was assessed indirectly by measuring PTINR. The mean changes from baseline in PT-INR were not higher in sorafenib patients compared to placebo patients, suggesting that sorafenib did not inhibit warfarin metabolism *in vivo*. However, patients taking warfarin should have their INR checked regularly.

- **CYP isoform-selective substrates:** Concomitant administration of midazolam, dextromethorphan and omeprazole, which are substrates of cytochromes CYP3A4, CYP2D6 and CYP2C19, respectively, following 4 weeks of sorafenib administration did not significantly alter the exposure of these agents. This indicates that sorafenib is neither an inhibitor nor a clinically meaningful inducer of these cytochrome P450 isoenzymes.

- **Combination with other anti-neoplastic agents:** In clinical studies, sorafenib has been administered together with a variety of other antineoplastic agents at their commonly used dosing regimens, including gemcitabine, oxaliplatin, doxorubicin, and irinotecan. Sorafenib had no effect on the pharmacokinetics of gemcitabine or oxaliplatin. Concomitant treatment with sorafenib resulted in a 21% increase in the AUC of doxorubicin. When administered with irinotecan, whose active metabolite SN-38 is further metabolized by the UGT1A1 pathway, there was a 67-120% increase in the AUC of SN-38 and a 26-42% increase in the AUC of irinotecan. The clinical significance of these findings is unknown. However, caution is recommended when administering sorafenib with doxorubicin and with compounds that are metabolized/eliminated predominantly by the UGT1A1 pathway (e.g. irinotecan).

Warfarin: Infrequent bleeding events or elevations in the International Normalized Ratio (INR) have been reported in some patients taking warfarin while on sorafenib therapy.

Patients taking warfarin concomitantly should be monitored regularly for changes in prothrombin time, INR and for clinical bleeding episodes.

7.4 Radiation

Initiation of conformal radiotherapy will be considered day 1 of study treatment. Treatment will consist of a total dose of 50 Gy (delivered in 25 fractions of 200 cGy over approximately 35 days). If treatment falls on a weekend or holiday, treatment can resume the next business day. The target volume of radiation therapy will include the site of the primary lesion and those tissues suspected of involvement by microscopic disease to a clinically important probability. Physical exam in conjunction with MRI and/or CT scans obtained during evaluation will be used to define the target volume. Treatment planning will aim to include a radial margin of approximately 2 cm and a longitudinal margin of approximately 3 cm beyond the gross disease.

Optimal field arrangement, beam parameters, and shaped blocks will be used to achieve the closest approximation of treatment volume to target volume to minimize irradiation of uninvolved normal tissue. Immobilization devices will be used daily to ensure reproducibility of treatment protocols. All possible efforts will be made to avoid treating the full circumference of an extremity or treating the lung. Patients may receive post-operative brachytherapy or external beam radiation therapy for positive microscopic surgical margins. Boost will not be given for patients with 100% necrosis. If indicated, RT may begin approximately 2 weeks following surgical resection, assuming there is satisfactory healing of the surgical wound. The target volume for the post-operative RT will be the tumor bed as defined by the operative and pathological findings.

7.5 Surgery

Surgical resection will take place on approximately day 50 – 90 of the study, occurring approximately 3 to 7 weeks following completion of combined neoadjuvant sorafenib/RT (**see study calendar, section 10.1**).

Surgical treatment necessary to resect the tumor with negative margins will be used. Biopsy sites will be excised en bloc with the definitive surgical specimen. The goal of surgical resection is to remove a 2 cm margin of normal tissue around the tumor without compromising function. Surgical dissection will take place through normal tissue planes and, where technically possible, will be done through a fascial plane outside the tumor. If the tumor is close to or displaces major vessels or nerves, these structures will be preserved if the adjacent adventitia or perineurium can removed and the margin is not involved pathologically. If postoperative pathologic evaluation reveals positive soft-tissue margins other than bone, nerve, or large blood vessels, this margin should be re-resected if possible. If bone, major blood vessel, or nerve is microscopically positive, additional radiation may be given as noted above. In general, lymph node dissection will not be performed, but a sampling can be performed if the primary tumor is over a major node station. Closed wound suction drainage should be used in all anatomic regions to evacuate postoperative seroma fluid. The drains should exit the skin close to the edge of the surgical incision. External

compression for extremity resections with ace wraps or compression dressings is also advised. Because all patients will have had radiation, special care must be given to skin flaps. Use of muscle flaps, pedicled myocutaneous flaps, and even free flaps is encouraged to fill dead space, particularly if there is any concern about the viability of the wound flaps. In general, the following principles should be followed in post-operative management of these patients:

- Maintain staples or skin sutures per surgeon preference but because of potential delay in wound healing, 3-4 weeks is recommended
- Leave drains until the drainage meets the criteria for surgeon preference for discontinuation
- Begin rehabilitation slowly

Resectability will depend upon the judgment of the operating surgeon. For the extremities, resection must be limb salvage procedure. Extremity patients who are not resectable without amputation may be amputated. Unresectable tumors may be palliated with surgery, chemotherapy, or additional radiation therapy. These patients will be taken off protocol.

Note that documentation of wound complications is an integral part of this clinical trial. Wound complications are defined in section 10.4.2 and must be accurately recorded and reported.

7.6 Pathologic Evaluation

All pathology slides from the initial diagnostic biopsy and the post-treatment surgical resection must be submitted for central review. Following combined neoadjuvant sorafenib/RT treatment, resected specimens will be examined carefully for determination of percentage tumor necrosis

Routine tissue sections will be analyzed for representative tumor histology and margin assessment. Approximately ten to fifteen tissue sections will be analyzed to ensure that an accurate overall assessment of tumor necrosis has been performed. The study pathologist will quantitate both the percentage of viable tumor and the percentage of tumor necrosis per slide. An overall assessment of percentage tumor necrosis for the entire tumor will then be calculated by summing the percent necrosis per individual slide divided by the total number of slides examined.

7.7 Duration of Therapy

Treatment should continue until the end of protocol therapy or until one of the following criteria applies:

- Disease progression,
- Intercurrent illness that prevents further administration of treatment,
- Unacceptable adverse event(s),
- Greater than 3-week delay in therapy due to adverse event,

- Patient presents with a positive beta-HCG test,
- Patient decides to withdraw from the study,
- General or specific changes in the patient's condition render the patient unacceptable for further treatment in the judgment of the investigator.

8.0 AGENT FORMULATION AND PROCUREMENT

8.1 Sorafenib (Nexavar)

Availability: Sorafenib will be supplied for this study

Product description: Sorafenib 200 mg is supplied as round, biconvex, red-film-coated tablets, debossed with the 'Bayer cross' on one side and '200' on the other side. The tablets contain sorafenib tosylate equivalent to 200 mg of the free base sorafenib, and the excipients croscarmellose sodium, microcrystalline cellulose, hypromellose, sodium lauryl sulfate, and magnesium stearate. The film-coat consists of hypromellose, polyethylene glycol, titanium dioxide and red iron oxide. The film coating has no effect on the rate of release of the active sorafenib tosylate. Study Drug can be supplied as sorafenib 200 mg commercial tablets in bottles of 140 tablets with a product identification label affixed or as commercial Nexavar in bottles of 120 tablets.

Storage requirements: Do not store above 25°C (77°F). Store in the original package.

Stability: The current shelf life is 36 months.

Route of administration: Orally.

Expected adverse events: The following additional drug-related adverse events and laboratory abnormalities were reported from clinical trials of sorafenib in 1286 cancer patients who received sorafenib as monotherapy (*very common* 10% or greater, *common* 1 to less than10%, *uncommon* 0.1% to less than 1%):
Cardiovascular**:** *Very Common*: hypertension *Uncommon:* hypertensive crisis, myocardial ischemia and/or infarction
Dermatologic: *Very common:* hand-foot skin reaction *Common:* , dry skin, pruritus *Uncommon:* folliculitis, , erythema multiforme
Digestive: *Very common:* anorexia, nausea, diarrhea, abdominal pain, weight loss *Common:* constipation, vomiting, hyperamylasemia, hyperlipasemia *Uncommon:* pancreatitis. Note that elevations in lipase are common; a diagnosis of pancreatitis should not be made solely on the basis of abnormal laboratory values
General Disorders: *Very common:* fatigue *Uncommon: hemorrhage*
Hematologic: *Common:* leukopenia, lymphopenia, thrombocytopenia *Uncommon:* INR abnormal
Hypersensitivity: *Uncommon:* hypersensitivity reactions (including skin reactions and urticaria)

Metabolic and Nutritional**:** *Very common:* hypophosphatemia *Common:* transient increases in transaminases *Uncommon:* hypothyroidism
Musculoskeletal: *Common:* arthralgia, myalgia
Reproductive**:** *Uncommon:* gynecomastia
Respiratory*: Uncommon:* hoarseness, rhinorrhea
In addition, the following medically significant adverse events were reported infrequently during clinical trials of sorafenib: cerebral hemorrhage, transient ischemic attack, cardiac failure, arrhythmia, thromboembolism, acute renal failure. For these events, the causal relationship to sorafenib has not been established.

9.0 CORRELATIVE/SPECIAL STUDIES

9.1 DCE-MRI

Subjects will undergo DCE-MRI scanning at baseline and at week 7 – 9. (approximately 2 – 4 weeks post-sorafenib/RT and approximately 1 – 3 weeks pre-surgery). Baseline DCE-MRI is to be performed 1 – 3 weeks before day 1 of treatment with sorafenib/RT.

DCE-MRI will be performed at the UC Davis Advanced Imaging Research Center. Following pilot scanning, DCE T_1-weighted MRI will be performed using a 3D fast spoiled-GRASS (FSPGR) pulse sequence to acquire over the time course 50 sets of images of the tumor. The other parameters are: TE = 2.3 ms, TR = 7.0 ms, 30^0 flip angle, 22 cm FOV, 256x64 matrix size. We expect each volumetric data set to contain about 25 image slices with 3 mm slice thickness, resulting in temporal resolution of 11.2 s for the time course data. Thus, the total scanning time for DCE MRI is expected to be less than 10 min. The Gd-based CR (0.1 mmol/kg dosage) will be delivered intravenously at 2 mL/s by a programmable power injector (Medrad, Indianola, PA) at the beginning of the third data set acquisition. The images of the first two data sets will be the baseline for the time courses. Conventional images will be taken with standard pulse sequences. We anticipate the total time in the scanner to be less than one hour.

Data will be transferred anonymously to off-line workstations and analyzed using the shutter-speed software package, which is written in the Matlab language. This program makes use of the two(or three)-water-site-exchange model and allows us to extract and pixel-by-pixel map pharmacokinetic parameters, such as K^{trans} v_e , and τ_i, the mean intracellular water molecule lifetime. The computation of the average K^{trans} and v_e and τ_i values from the lesion region-of-interest (ROI) and parametric mapping of K^{trans} and v_e and τ_i will be performed with both the FXL and FXR models.

9.2 Serum and Tissue Biomarkers

Exploratory molecular correlative analyses will be conducted on serial tumor and plasma specimens to identify biomarkers predictive of patient outcome and investigate sorafenib-induced changes in the circulation of pro-angiogenic factors and tumor signal transduction pathways (**see section 1.8 for study rationale**). Plasma will be collected for measurement of a panel of angiogenic markers that includes, but is not limited to, VEGF, OPN, PDGF, bFGF, IL-8 and IL-6 at baseline, 1 – 3 weeks after completion of combined neoadjuvant sorafenib/RT treatment, and then 1 – 3 weeks before surgical resection of the tumor (approximately 3 – 7 weeks following completion of sorafenib/RT). Blood samples (10 mL) will be drawn using venipuncture into a Vacutainer containing potassium EDTA and inverted gently several times to mix with anticoagulant. Within 10-15 minutes after collecting, blood samples will be centrifuged in a refrigerated (4°C) centrifuge for 10 minutes to separate the plasma. If a refrigerated centrifuge is not available, the tubes should be chilled in an ice bath for 5-15 minutes and then placed in a standard centrifuge for 10 minutes to separate the plasma. The plasma will be transferred to polypropylene tubes and frozen at -70°C or lower. The tubes will be labeled with the study number, and subject's study number, and time and date of the sample acquisition.

Fresh tumor tissue will be collected at baseline (pre-treatment with sorafenib/RT), and then at time of surgical resection on approximately study day 50 – 90. Tissue will be immediately frozen with liquid nitrogen and stored at –70°C or lower. Tumor tissue will be mechanically homogenated for 5 minutes on ice in 1 ml of extraction buffer per 100 mg tissue wet weight, and extract obtained after centrifugation for 20 minutes at 4°C will be transferred to polypropylene tubes and frozen at -70°C or lower. The tubes will be labeled with the investigator's name, study number, and subject's study number, and time and date of the sample acquisition. Multiplex analysis using Luminex technology and/or ELISA for VEGF, sVEGFR2, and bFGF will be performed on plasma and tumor extracts using commercial kits. Manufacturers' protocols will be followed and samples are to be measured in duplicate. Changes in activation states of MAPK signal transduction intermediaries (pERK) will be evaluated by Western blotting.

Paraffin block of the primary tumor biopsy and resected tumor will be obtained. Representative areas from each whole-mount paraffin block will be chosen as the best composite representation of tumor based on H&E morphology and will be placed in standard-sized tissue cassettes. These will be re-embedded, sectioned at 4 µm, and mounted on poly-L-lysine–coated slides for immunohistochemistry (IHC). Sections of paraffin embedded tissue will be deparaffinized and rehydrated. After appropriate antigen retrieval methods, staining with commercial antibodies for VEGF-R2, phospho-VEGF-R2, PDGFR, and phospho-PDGFR will be performed using an automated Dako immunostainer. Staining will be visualized by incubating the slides with 3,3'-diaminobenzidine solution, after which they will be rinsed, counterstained with hematoxylin, dehydrated in a Leica Autostainer XL, coverslipped, and reviewed by a single experienced pathologist who will score the percentage of positively staining cells. The activation status of pERK, pAKT, and

pmTOR will be evaluated by IHC using phosphor-specific antibodies established at UC Davis.

9.3 Function/Quality of Life Assessment

Functional status and quality of life will be assessed at baseline, during the rest period prior to surgery, and 30 days following surgery using standard metrics including the Toronto Extremity Salvage Score (TESS—appendix 14.1.3) and the SF-36 Health Survery (appendix 14.1.4).

10.0 STUDY CALENDAR/SCHEDULE OF EVENTS/STUDY PROCEDURES

10.1 Study Calendar

	**Before Study***	**Start of Study**<br><br>**Start of Rx**	**Rx**	**Rx**	**Rx**	**End of Rx***			**Resume Rx**	**End of Study/ Follow Up**
							**Rest**	**Rest**		
		Week 1	Week 2	Week 3	Week 4	Week 5	Week 6-8	Week 7-9	Week 8 - 12	
		**Day 1**							**Day 70**	
Informed Consent	☑									
Doctor's Visit, Measure BP	☑	☑	☑	☑	☑	☑	☑	☑	☑	☑
**Procedures**										
Blood Tests	☑		☑				☑	☑		☑
Pregnancy Test	☑									
CT/MRI Scan of Tumor	☑							☑		☑
CT Scan of Chest	☑							☑		☑
Biopsy of Tumor (Core/ Incisional)	☑									
Dynamic Contrast Enhanced MRI	☑							☑		
**Treatments**										
Sorafenib Tablets		☑☑☑☑ ☑☑	☑☑☑☑ ☑☑☑	☑☑☑☑ ☑☑☑	☑☑☑☑ ☑☑☑	☑☑☑☑ ☑☑☑				
Radiation Treatments		☑☑☑☑ ☑	☑☑☑☑ ☑	☑☑☑☑ ☑	☑☑☑☑ ☑	☑☑☑☑ ☑				
Surgery to Remove Tumor									☑	

* see below for timing of pre-study tests

10.2 Subject Registration

- Written informed consent must be obtained before any study specific medical procedures are performed.
- All patients must be registered with the UC Davis Cancer Center.
- Subjects being enrolled during the study will need to be assigned a dose level for treatment (see section 7.1).

10.3 Baseline Screening

- To be completed within 6 weeks of registration:
  CT of Chest, MRI/CT of primary tumor location
- To be completed within 4 weeks of registration:
  History & Physical, including vitals, PS
  EKG
  Labs
- To be completed within 1 week of registration:
  Serum or urine pregnancy test

10.4 Study Visits

See study calendar (section 10.1)

10.4.1 Toxicities and Adverse Events

Toxicities and adverse events will be assessed at each visit using the NCI Common Toxicity Criteria for Adverse Events v3.0 (http://ctep.cancer.gov/reporting/ctc.html). **Acute and long-term radiation toxicity must be graded at each visit for the following CTCAE categories:**
1) Dermatologic/Rash: Dermatitis associated with radiation
2) Musculoskeletal/Soft Tissue: Extremity (as appropriate for location of tumor)
3) Musculoskeletal/Soft Tissue: Fibrosis—deep connective tissue

10.4.2 Assessment of Wound Complications

An accurate assessment of post-operative wound complications is vital for the accurate reporting of toxicity associated with this regimen. Major wound complications will be defined as described by O'Sullivan et al. in the randomized, controlled trial of preoperative versus postoperative radiation in the treatment of STS of the extremities.[8]

A major wound complication is defined as a secondary operation under general or regional anesthesia for wound repair (debridement, operative drainage, and secondary wound closure including rotationplasty, free flaps, or skin grafts), or wound management without secondary operation. Wound management includes an invasive procedure without general or regional anesthesia (such as aspiration of seroma), readmission for wound care such as intravenous antibiotics, or persistent deep packing for 120 days or longer.

10.4.3 Follow-up

Follow-up for all patients registered on this study, including those who do not receive any protocol therapy, will be required for 5 years following completion of therapy. Follow-up after completion of protocol therapy will be limited to monitoring for 1) development of local tumor recurrence, 2) development of distant metastatic disease, 3) survival, and 4) late toxicity, including limb function impairment and secondary malignancy. Follow-up reports are due every 4 months.

10.4.4 Schedule of Events

	Prior to Registration	Prior to Day 1 of Treatment	Time of Pre-Surgical Restaging*	Post Treatment Follow-up
**Tests & Observations**				
History and Progress Notes	X	X	X	X
Physical Examination	X	X	X	X
Pulse, Blood Pressure**	X	X	X	X
Height/Weight	X			
Performance Status	X	X	X	X
Treatment Toxicity Assessment		#	#	#
Pathology Review	##		##	
**Laboratory Studies**				
CBC, Differential, Platelets	X		X	PRN
Serum Chemistries***	X		X	PRN
Pregnancy Test****	X			
INR	X	###	###	
EKG	X			PRN
DCE-MRI		See section 9.1	See section 9.1	
Tumor tissue/serum for correlative studies		See section 9.2	See section 9.2	
**Staging**				
Chest CT	X		X	####
MRI or CT of primary site				####

* After completion of combined sorafenib/radiotherapy and within 4 weeks of surgery.

** Blood pressure to be monitored weekly during treatment and study period until time of surgery.

*** Comprehensive metabolic panel (including BUN, creatinine, bilirubin, AST, ALT, alkaline phosphatase).

**** For women of childbearing potential.

\# Toxicity assessments per CTCAE v 3.0 (See section 11.4.1 and appendix B). Long term follow-up for radiation toxicity and limb function required per section 11.4.1.

Initial diagnostic pathology specimen must be submitted for central review. Post-resection specimen will be carefully evaluated for percent tumor necrosis following preoperative treatment (see section 7.5).

INR should be closely monitored for patients receiving warfarin. INR does not need to be monitored during study for patients not receiving warfarin.

Post-treatment imaging studies for disease recurrence is at investigator's discretion. It is suggested that a chest CT or chest X-ray be performed every 4 months for the first 2 years, every 6 months for the 3rd year, and yearly for the 4th and 5th year. It is suggested that MRI or CT of the primary site be obtained yearly for the first 3 years.

10.5 Specimen Submission for Molecular Correlative Studies

With the patient's consent, tissue and blood specimens will be submitted as outlined below.

- Paraffin-embedded tissue blocks or slides from the time of diagnosis (prior to therapy) and at the time of surgical resection will be submitted for expression of relevant molecular targets. 1-2 paraffin-embedded tissue blocks containing formalin-fixed tumor tissue should be submitted for evaluation and expression of mutations in relevant molecular pathways. Paraffin blocks should be processed according to standard institutional protocols. If blocks are unavailable, 16 unstained slides are acceptable alternatives.

- Two blood specimens will be collected from each patient prior to treatment (2 x 10 mL). Subsequent blood specimens (1 x 10 mL) will be collected at selected intervals as indicated in the study calendar (see section 10.1, weeks 2, 6, 9, and end of study). In the event that a patient is removed from the study, a blood specimen (approximately 10 mL) will be obtained at the time the patient is removed from protocol treatment.

One blood specimen should be collected in a 10 mL purple-top (EDTA) tube, inverted several times, and placed on wet ice until centrifugation. The tube should be centrifuged as soon as possible at approximately 1,000 to 1,500 rpm for 10 minutes. Plasma should be removed and placed in 1 mL aliquots in labeled cryotubes. Buffy coat cells should be separately removed and placed in labeled cryotubes. All specimens must be labeled with protocol number, patient registration number, and dte of specimen collection. All tubes are then to be frozen (snap frozen with liquid nitrogen if possible) and stored at -70°C until shipped to the laboratory on dry ice as detailed below.

The second blood specimen should be collected in a 10 mL red-top tube, allowed to clot at room temperature for 30 minutes, and placed on wet ice until centrifugation. The tube should be centrifuged at 3000 rpm for 10 minutes. Serum should be removed and placed in 1 mL aliquots in labeled cryotubes. All specimens must be labeled with protocol number, patient registration number, and date of specimen collection. All tubes are then to be frozen (snap frozen with liquid nitrogen if possible) and stored at -70°C until shipped to the laboratory on dry ice as detailed below.

- Shipping instructions: All archival paraffin block or slide specimens should be sent at ambient temperature. Frozen specimens should be shipped on dry ice. These should be shipped by overnight courier Monday through Wednesday only, to the following address:

  Philip C. Mack, PhD
  Suite 3016, Division of Hematology/Oncology
  UC Davis Cancer Center
  4501 X Street
  Sacramento, CA 95817

  [redacted]

A Specimen Submission Form must be submitted with each specimen. Institutions should notify the recipient by either phone or fax prior to shipping specimens. This will allow the recipient to track the package in the event that there are any problems in delivery.

The Federal Guidelines for Shipment are as follows (these periodically change, please check for the most current guidelines):
1. The specimen must be wrapped in an absorbable material
2. The specimen must then be placed in an AIRTIGHT container (resealable bag)
3. Pack the resealable bag and specimen in a styrofoam shipping container
4. Pack the styrofoam shipping container in a cardboard box
5. The cardboard box should be labeled “UN3373 Biological Substance, Category B “BIOHAZARD”

11.0 MEASUREMENT OF EFFECT

11.1 Pathologic Response

This is the primary endpoint of the phase II trial. **(see section 7.5 for protocol-based determination of this endpoint)**

11.2 Radiographic and Clinical Response

The radiographic and/or clinical response of the primary tumor to preoperative therapy will be evaluated. The primary lesion will serve as the sole target lesion and will be recorded and measured at baseline. The longest diameter (LD) of the target lesion will be measured and reported as the baseline LD. The baseline LD will be used as reference to further characterize the objective tumor response of the measurable dimension of the disease.

Response and progression will be evaluated in this study using the new international criteria proposed by the Response Evaluation Criteria in Solid Tumors

(RECIST) Committee.[57] Changes in only the largest diameter (unidimensional measurement) of the tumor lesions are used in the RECIST criteria.

11.3 Response Criteria

11.3.1 Evaluation of target lesion

Complete Response (CR): Disappearance of target lesion

Partial Response (PR): At least a 30% decrease in the sum of the longest diameter (LD) of target lesion, taking as reference the baseline sum LD

Progressive Disease (PD): At least a 20% increase in the sum of the LD of target lesion, taking as reference the smallest sum LD recorded since the treatment started or the appearance of one or more new lesions

Stable Disease (SD): Neither sufficient shrinkage to qualify for PR nor sufficient increase to qualify for PD, taking as reference the smallest sum LD since the treatment started

11.3.2 Evaluation of best overall response

The best overall response is the best response achieved prior to surgical resection of the target lesion.

Target Lesions	New Lesions	Overall Response
CR	No	CR
PR	No	PR
SD	No	SD
PD	Yes or No	PD
Any	Yes	PD

NOTE: In some circumstances, it may be difficult to distinguish residual disease from normal tissue. When the evaluation of complete response depends upon this determination, this will be determined at the time of surgical resection.

11.4 Time-to-Event Measures

11.4.1 Time to Local Recurrence

Defined as the duration of time from surgical resection of the primary tumor until local recurrence (amputated patients excluded).

11.4.2 Local Disease-Free Survival

Defined as the duration of time from surgical resection of the primary tumor until local recurrence or death, whichever occurs first.

11.4.3 Distant Disease-Free Survival

Defined as the duration of time from registration until development of distant metastatic disease or death, whichever occurs first.

11.4.4 Disease-Free Survival

Defined as the duration of time from surgical resection to local recurrence, distant metastatic disease, or death, whichever occurs first.

11.4.5 Progression-Free Survival

Defined as the duration of time from registration to progressive disease, local recurrence, distant metastatic disease, or death, whichever occurs first.

11.4.6 Overall Survival

Defined as the interval of time from registration until death from any cause.

12.0 ETHICAL AND REGULATORY REQUIREMENTS

12.1 Protocol Review

The protocol and informed consent form for this study must be reviewed and approved in writing by the UC Davis Scientific Review Committee (SRC) and the Institutional Review Board (IRB) prior to any patient being registered on this study.

12.2 Informed Consent

Written informed consent will be obtained from all patients, or the legally authorized representative of the patient, participating in this trial, as stated in the Informed Consent section of the case of Federal Regulations, Title 21, Part 50. If a patient’s signature cannot be obtained, the investigator must ensure that the informed consent is signed by the patients legally authorized representative. Documentation of the consent process and a copy of the signed consent shall be maintained in the patient’s medical record.

12.3 Protocol changes or amendments

Any modification of this protocol must be documented in the form of a protocol revision or amendment signed by the principal investigator and approved by the SRC and the IRB, before the revision or amendment may be implemented. The only circumstance in which the amendment may be initiated without regulatory approval is for a change necessary to eliminate an apparent and immediate hazard to the patient. In that event, the investigator must notify the SRC and the IRB in writing within 10 working days after the implementation.

12.4 Maintenance of Records

If the investigator relocates or for any reason withdraws from the study, then the study records must be transferred to an agreed upon designee, such as another institution, another investigator, or to the UC Davis Clinical Trials Support Unit (CTSU). Records must be maintained according to sponsor and FDA requirements.

12.5 IRB Reporting of Unanticipated Problems and Adverse Events

Unanticipated Problems (UP) and Adverse Events (AE) will be reported to IRB according to the policies, procedures and guidelines posted on the IRB web site http://www.research.ucdavis.edu/home.cfm?id=ovc,1,1301. Fatal and life-threatening UP will be reported to IRB within 7 days of notification of the event. All other UP reports will be submitted to the IRB no later than 15 days after occurrence or notification of the event. Copies of the report documents will be kept in the study regulatory binder.

12.6 MedWatch Reporting

For this investigator-initiated study, the investigator is considered the sponsor. The investigator/sponsor is required to report adverse experiences to the FDA through the MedWatch reporting program, even if the trial involves a commercially available agent. Adverse experiences to be reported include any unexpected (not listed in the package label), serious adverse experiences with a suspected association to the study drug. These adverse experiences will be reported using a MedWatch form 3500 for voluntary reporting. MedWatch forms and instructions are available at www.fda.gov/medwatch . MedWatch reports can be submitted online at https://www.accessdata.fda.gov/scripts/medwatch/

When the serious adverse event is reported to the FDA, copies of the MedWatch 3500 form and supporting materials will be submitted to the UC Davis IRB and the UC Davis CTSU. A copy of the MedWatch 3500 form and supporting materials will be kept on file in the study regulatory binder.

12.7 Inclusion of Women, Minorities and Children

12.7.1 Inclusion of Women and Minorities

This study will **not** focus on any particular gender, racial or ethnic subset. **No subject will be excluded from the study on the basis of gender, racial, or ethnic origin.** Male, female and minority volunteers will be recruited for this study from the general population and approximately 50% men and 50% women will be studied. The projected gender, racial, and ethnic composition of the study will represent that of the state of California.

12.7.2 Inclusion of Children

In accordance with NIH guidelines on the inclusion of children as participants in research involving human subjects, children under the age of 18 years must be included in all human subjects research, conducted or supported by the NIH, unless there are clear and compelling reasons not to include them. Therefore, proposals for research involving human subjects must include a description of plans for the inclusion of children.

This protocol does not include children < 18 years of age for the following reason: pediatric soft tissue sarcomas (e.g. embryonal rhabdomyosarcoma, Ewing's sarcoma, etc…) have established and clinically effective chemotherapeutic protocols which have been proven to favorably impact outcome and prognosis in the adjuvant/neoadjuvant setting. Enrolling children < 18 into this investigational protocol would therefore potentially prevent them from receiving established first-line therapy.

13.0 STATISTICAL CONSIDERATIONS

13.1 Primary Objective

13.1.1 Phase I Trial

To determine the maximally tolerated dose (MTD) of the antiangiogenic agent sorafenib in combination with preoperative conformal radiotherapy for soft tissue sarcomas of the extremities and the body wall, high grade > 5 cm in diameter and low grade > 8 cm. A traditional dose-escalation design will be used to determine the MTD of sorafenib combined with radiotherapy. 3-6 patients will be evaluated at each of 2 dose levels, based on a starting dose of 200 mg bid derived from preclinical studies of sorafenib combined with radiotherapy.[32, 33] Since 400 mg bid is the well established MTD for sorafenib monotherapy in patients with renal cell carcinoma and hepatocellular carcinoma,[45, 46] the dose will not be escalated above this level even if DLT is not observed.

MTD is defined as the dose that produces dose limiting toxicity (DLT) in 33% of patients. Dose level escalation will be determined based on DLTs observed through the first 70 days of therapy, but DLTs will be monitored in the post-operative period. Dose de-

escalation may occur if excess late DLTs are observed. **See Section 7.1 for further explanation of determination of DLT .** Briefly, if 0/3 patients experience DLT, the dose will be escalated for the next cohort of 3 patients. If 1/3 patient experience DLT, the same dose will be repeated for the next cohort of 3 patients. If 2/3 or 3/3 patients experience DLT, the study will be terminated or dose reduced. Toxicity and wound complication rates will be reported.

13.1.2 Phase II Trial

The primary endpoint of the phase II trial will be the rate of complete/near-complete pathologic response (≥ 95% tumor necrosis) following treatment with this multimodality therapy. Anticipating an improvement in the pathological complete/near-complete response rate from 10% with RT alone to 33% with combined sorafenib and RT, we will have an 80% power to detect this difference if we accrue 25 patients, assuming a type I error rate of 5%.

Treatment Stop Rule

A pause in study accrual will be implemented in the phase II portion of the trial after 10 patients have completed concurrent sorafenib plus conformal radiotherapy followed by surgical resection. Safety assessment will be made 6 weeks following resection of the 10th patient (9 – 13 weeks following completion of concurrent sorafenib and RT) to ensure that excess DLTs are not observed. The study will be ***terminated for safety*** (not lack of efficacy) if 4 of these 10 patients experience grade 3 adverse events or greater since this would represent an increase from the baseline rate of serious AEs expected from the standard combination therapy of preoperative radiation and surgical resection.[8] This pause in accrual will allow us to review all safety data collected to date. If DLTs in excess of our stopping rule are not observed, then the phase II trial will continue to the targeted accrual of 25 patients.

13.2 Secondary Objectives

13.2.1 To determine the rate of R0 (negative resection margin), R1 (microscopically positive resection margin), and R2 (macroscopically resection margin) following neoadjuvant conformal radiotherapy in combination with sorafenib.

13.2.2 To analyze changes in tumor blood flow, blood volume, capillary permeability, and cell viability pre- and post-treatment with combined sorafenib/RT using dynamic contrast-enhanced MRI. The primary endpoints of these studies will be the transfer constant (Ktrans) and the initial area under the gadolinium concentration time curve (IAUGC, mMGd◦min—**see section 9.1 for DCE-MRI imaging protocol)**.

To account for within-subject correlation, we will use a mixed effects model to analyze changes in DCE-MRI pharmacokinetic parameters (K^{trans} and v_e) at baseline and then following neoadjuvant sorafenib/RT. These

comparisons will be made within the framework of the mixed effects model. Galbraith et al.[58] reported the mean (range) to be 0.43 ml/ml/min (0.07-1.03) for K^{trans} and 32.2 ml/ml (12.9-62.4) for v_e among human tumors. If we assume that the range represents ± 2 SD, SD is approximately 0.24 for K^{trans} and 12.38 for v_e. A sample size of 25 patients will provide 71% power to detect a 1.0 standard deviation change in K^{trans} and v_e between these two time points.

13.2.3 To initiate analyses of molecular markers of tumor response assessment comparing (1) a microarray of a panel of serum angiogenic markers pre- and post-treatment; and (2) immunohistochemistry of tumor tissue pre- and post-treatment for expression of angiogenic proteins (VEGF, VEGFR1-3), growth factors correlated with hypoxia and resistance to radiation (EGF, FGF, HIF-1α), and modulators of proliferation and signal transduction (Ki-67, pERK). These analyses will be conducted in an exploratory manner with the aim of generating clinically and biologically interesting hypotheses. We will correlate pre- and post-treatment tissue and plasma biomarker levels (as well as the pre- and post-treatment changes) with DCE-MRI parameters and clinical outcomes. A regression analysis will be used to assess a correlation with DCE-MRI parameters. Logistic regression analysis will be used for binary clinical outcomes (e.g., ≥ 95% necrosis) and Cox regression analysis will be used for time-to-event outcomes.

13.2.4 To obtain preliminary data regarding local disease control, distant disease control, progression-free survival, and overall survival with this novel therapeutic regimen. Kaplan-Meier method will be used to estimate time-to-event measures. For definitions of time-to-event measures, see Section 11.4

13.2.5 To determine the rate of radiographic response to neoadjuvant sorafenib/RT. Cross-sectional radiographic response will be evaluated by RECIST criteria **(see section 11.3)**. Categorical differences in these data (complete response, partial response, stable disease, and progressive disease) will be tested against pathologic response categories using the Chi-squared/Fisher's exact test.

14.0 APPENDICES

14.1.1 ECOG Performance Status

0	Asymptomatic and full active
1	Symptomatic; fully ambulatory; restricted in physically strenuous activity
2	Symptomatic; ambulatory; capable of self-care; more than 50% of waking hours are spent out of bed

3 Symptomatic; limited self-care; spends more than 50% of time in bed, but not bedridden
4 Completely disabled; no self-care; 100% bedridden

14.1.2 IRAT DCE-MRI Protocol

Imaging Response Assessment Team Protocol for DCE-MRI

Protocol Adapted from Recommendations of 2004 NCI Workshop

Natarajan Raghunand, Ph.D., University of Arizona, Tucson
Michael H. Buonocore, MD, Ph.D., University of California, Davis

Overall

- Magnet Strength 1.5 T.
- Low molecular weight gadolinium chelates which remain extracellular, e.g. Magnevist® or Omniscan®.
- The reconstructed image data must be saved in a quantitative manner – i.e., all scaling factors must either be factored out before saving, or a uniform scaling factor applied to images which are to be compared to one another. This includes both factors which affect the signal strength during acquisition (e.g., "Receiver Gain"), and factors applied when saving of the image data in 16-bit integer or other format.

Pre-injection

- Acquire high quality clinical images of entire anatomic region (preferably in two orthogonal planes).
- Acquire T1- and T2-weighted images registered in the same planes as the dynamic data.
- Slice planning: Try to include the cross-section of the descending aorta (reference artery) in the field-of-view. Locate target slices under both "Held Inspiration" breath-hold and "Held Expiration" breath-hold. Bony markers may be used for this purpose (i.e., assuming "rigid-body motion"). Very Important: If this is the post-treatment scan of a subject, then slices should be positioned as closely as possible to match the slices in the pre-treatment scans of that patient.
- T1 Map: It is strongly recommended that a pre-contrast T1 map be acquired using the final slice geometry.

Contrast agent injection

- Use power injector to minimize variation in injection kinetics.
- Injection dose should be standardized by weight (0.1 mmole Gd/Kg).
- 15 sec for total injection, followed by minimum 20 cc saline flush.
- Document injection site, use same site for subsequent studies.
- Minimum of 24 h between studies on a given patient.

Dynamic Portion of the Study

- "Held Expiration" (HE) is more reproducible and less prone to motion related to air leakage than "Held Inspiration" (HI). The dynamic images are therefore acquired during HE. As already mentioned, scout scans should be reacquired under HE, if necessary.
- Imaging parameters: There is flexibility here, but one suggested sequence might be a GRE sequence with TR=43 ms, TE=minimum, alpha=50 degrees, 256x160 matrix. Fat-suppression must be used. The example scan durations given in the next section are for these parameters.
- DCE Imaging:
  - Instruct the patient to "breathe in, breathe out, breathe in, breathe out, and hold", and acquire two pre-Gd images during this first HE (2 x 7 s per repetition = 14 seconds).
  - Instruct the patient to "breathe in, breathe out, breathe in, breathe out, and hold", begin injection, and acquire two images (2 x 7 s per repetition = 14 seconds).
  - "Breathe in, Breathe out, Hold, Scan" (7 s scantime plus time to breathe in and breathe out ≈ 17 seconds temporal resolution, depending on patient).
  - Repeat "Breathe in, Breathe out, Hold, Scan" through 4 min post-injection. Monitor pulseOX to see if patient is tolerating the repeated breath-holding. If not, then switch to next step immediately.
  - Around 4 min post-injection, switch to "Breathe in, Breathe out, Breathe in, Breathe out, Hold, Scan". This increases temporal resolution to around 24 seconds per scan, depending on the patient.
  - Continue scanning out to 8 min post-injection.

Primary endpoints

- The primary endpoint should be the transfer constant (K^{trans}, min^{-1}) and/or initial area under the gadolinium concentration time curve (IAUGC, mMGd·min, over the first 90 seconds after contrast arrival).
- Measurements of K^{trans} or IAUGC should be made voxelwise.

- In tissues with substantial motion ROI average measurements may be appropriate.
- A minimum of 3 slices are preferred as single slice measurements are more prone to bias.
- Tumor dimensions (three orthogonal axes) for total tumor and region analyzed should be reported.
- Vascularized tumor volume can be obtained by summing voxels with values above a predetermined threshold (report threshold definition).
- All data including ROI definition and analysis should be recorded and traceable to support external review.

Measurement requirements to assess K^{trans} and IAUGC

- Both K^{trans} and IAUGC require calculation of instantaneous tumor gadolinium concentration, based on the change in relaxation time due to contrast uptake ΔR1. This requires:
  - An estimate of contrast agent relaxivity
  - Measurement of tumor T1 immediately prior to contrast uptake
  - An accurate T1 measurement method verified for all spatial locations, coils and scanners used
  - Arterial Input Function (or normalization function) (or Cardiac Output)
  - Reproducible injection

Secondary endpoints

- Other endpoints derived from compartmental models and DCEMRI such as V_b, V_e, K_{ep} may be of value
- Simplified methods based on signal intensity changes may be less sensitive than K^{trans} or IAUGC and are harder to compare between centers
- More elaborate pharmacokinetic models may improve evaluation of dynamic data but are not yet supported by sufficient evidence to warrant use as primary endpoints

Trial design

- Entry criteria should consider tumor size in relation to pharmacological mechanisms, MRI resolution, sensitivity to motion and potential confounding factors from previous treatment (e.g., radiation) or rapid tumor growth rates

- Tumors in a fixed superficial location should be at least 2 cm in diameter; other tumors should be at 3 cm in diameter

Nomenclature

- Standardized terms should be employed as defined in Tofts et al (JMRI, 10:223-232, 1999)

Analysis

- Model analysis should be based on the well-accepted Tofts or equivalent models, but with inclusion of arterial input normalization (or equivalent normalization function), blood volume, and classification of fit failures
- Estimates of uncertainty should monitor model fitting and chi-squared error, mapping this factor and including it in error analysis
- Fit failures should be categorized as model fit failure (possibly multiple classes), no enhancement, or noise
- ROI analysis, based on whole tumor mean values, may not evaluate tumor heterogeneity; although it may be robust to motion, it may not reflect small areas of rapid change and so may be insensitive
- Voxel mapping allows all data to be evaluated, allowing description and evaluation of regional change; individual voxels will have relatively poor signal to noise
- Analysis techniques, such as histogram and principal components analysis, may yield sensitive assessment of change.

ROI

- ROI placement needs to be supported by method of definition, and recorded to permit re-evaluation.
- Before placement of an ROI, individual images should be examined for the presence of patient motion, best seen on subtraction images
- Ideally dynamic image datasets should be spatially registered before analysis
- Both early (60-120sec after contrast) and late (more than 5 min after contrast) subtraction images should be generated
- Ideally the early subtraction images may guide the position for ROI placement (may not apply in some region; e.g., liver), which should also take account of non-enhanced images (i.e., include non-enhancing tumor)
- If early enhancement is low, the late subtraction dataset should be used

- If no enhancement is seen, the baseline data (non-enhanced) aided by conventional images should be used for ROI placement
- The outer limit of the lesion should act as a boundary of the ROI to minimize partial volume effects.
- Adjacent blood vessels or regions with other sources of artefact should be excluded
- The ROI should be constant in position and size for each image in the series under analysis; in the event of significant motion, it may be necessary to adjust the ROI position on each image, measuring only a mean value
- The position of the ROIs, corresponding graphs and table of enhancement values should be recorded, ideally in digital and hard copy form for future reference
- Analysis should take account of potential partial volume and ROI shape
- Definition of ROI should be stated explicitly in all reports of DCE-MRI data

Specific Implementation of the above DCE-MRI protocol on a GE EchoSpeed 1.5T MRI System

This EXAMPLE PROTOCOL follows the general guidelines listed above, except that for the dynamic contrast enhancement scan it uses a fast GRE sequence with approximately one second acquisition per slice, rather than the standard GRE sequence with a 7 second acquisition per 2 slices. Consequently, the acquisition and breathing instructions are different from the guidelines listed above (unless the fast GRE sequence is used to acquire 7 slices per breathhold).

The parameter listings below are the basic parameters excerpted from the protocol definition files of the MRI system. Complete files are available on request. In the parameter listings below, "" with no value given means that the corresponding parameter value is set automatically by MRI system. Glossary of parameter names is provided at the end of this section.

Common parameters for entire study:

```
set ENTRY "Feet First"
set POSITION "Supine"
set COIL "TORSOPA"
```

1. localizer

```
set PLANE "3-PLANE"
set SEDESC "3 plane loc"
set IMODE "2D"
set PSEQ "Localizer"
set TE ""
set NECHO "1"
set TR ""
set FOV "40"
set SLTHICK "10.0"
```

```
set SPC "4.0"
set SL3PLANE "11"
set NOSLC "11"
set MATRIXX "256"
set MATRIXY "128"
set SWAPPF "Unswap"
set NEX "2.00"
set PHASEFOV "1.00"
set AUTOCF "Water"
set RBW ""
set AUTOSHIM "Yes"
```

Tech notes: Localizer FOV will be 40-48 cm. This is pre-contrast, free-breathing scan.

2. 2D FSE T2 (Coronal):

```
set PLANE "CORONAL"
set SEDESC "FSE T2 Fatsat"
set IMODE "2D"
set PSEQ "Spin Echo"
set IOPT "Fast"
set PSDNAME "fse-xl"
set SATTHICKZ1 "80.0" (Superior SAT)
set SATTHICKZ2 "80.0" (Inferior SAT)
set TE "50.0"
set NECHO "1"
set TR "3000.0"
set ETL "8"
set FOV "32"
set SLTHICK "8.0"
set SPC "1.0"
set NOSLC "24"
set MATRIXX "256"
set MATRIXY "256"
set SWAPPF "R/L" (sets frequency encode direction)
set NEX "1.00"
set PHASEFOV "1.00"
set AUTOCF "Water"
set RBW "31.25"
set AUTOCF "Water"
```

Tech note: Set for at least 24 slices (minimum). Cover the entire liver plus margin to allow localization. This is pre-contrast, free-breathing scan. In this scan and all subsequent scans, FOV and number of slices can be changed to match patient girth and organ dimensions.

3. 2D FSE T2 (Axial T2):

```
set PLANE "AXIAL"
set SEDESC "FSE T2 Fatsat"
set IMODE "2D"
set PSEQ "Spin Echo"
set IOPT "Fast"
set PSDNAME "fse-xl"
set SATTHICKZ1 "80.0" (sets superior SAT)
set SATTHICKZ2 "80.0" (sets inferior SAT)
```

```
set TE "50.0"
set NECHO "1"
set TR "3000.0"
set ETL "8"
set FOV "32"
set SLTHICK "8.0"
set SPC "1.0"
set NOSLC "24"
set MATRIXX "256"
set MATRIXY "256"
set SWAPPF "R/L" (sets R/L frequency encode direction)
set NEX "1.00"
set PHASEFOV "1.00"
set AUTOCF "Water"
set RBW "31.25"
```

Tech notes: Set for at least 24 slices (minimum). Cover the entire organ plus margin to allow localization. This is pre-contrast, free-breathing scan.

4. Pre-infusion T1 (Fast SPGR, 18 slices)

```
set PLANE "AXIAL"
set SEDESC "FSPGR PRE TE 1.8 All-slice"
set IMODE "2D"
set PSEQ "SPGR"
set IOPT "VBw, EDR, Fast"
set FLIPANG "60"
set TE "Out of Phase" (sets TE to 1.8)
set NECHO "1"
set TR "100.0"
set FOV "32"
set SLTHICK "8.0"
set SPC "1.0"
set NOSLC "18"
set MATRIXX "256"
set MATRIXY "160"
set SWAPPF "R/L"
set NEX "1.00"
set PHASEFOV "1.00"
set AUTOCF "Water"
set RBW "15.63"
```

Tech notes: Slices should extent across entire lesion plus a margin (e.g., 1 cm). To obtain a subset of slices obtained in the FSE sequence (Series 2), in the Graphics screen the FSE slices are first copied as a group, then a second slice group is formed and overlaid on the FSE group at the desired locations. Be sure to line up the slice centers as well as the lines themselves. After the second group is defined, the FSE group is highlighted and erased. Fat sat is turned on. This is pre-contrast, free breathing scan. Need to record the receiver gain for scan #9. However, the receiver gain can be retrieved from the Text page window in the Image Browser. In this scan and all subsequent scans, FOV can be changed to match patient girth.

5. Pre-infusion T1 (Fast SPGR, multiphase, 10 acquisitions, 3 slices)

```
set PLANE "AXIAL"
set SEDESC "FSPGR Pre FA30 10 acq 3-slice"
```

```
    set IMODE "2D"
    set PSEQ "SPGR"
    set IOPT "Seq, VBw, EDR, Fast, MPh"
    set SLPERLOC "10"
    set DELACQ "Minimum"
    set FLIPANG "30"
    set TE "Out of Phase" (sets TE 18)
    set NECHO "1"
    set FOV "32"
    set SLTHICK "8.0"
    set SPC "1.0"
    set NOSLC "3"
    set MATRIXX "256"
    set MATRIXY "160"
    set SWAPPF "R/L"
    set NEX "1.00"
    set PHASEFOV "1.00"
    set AUTOCF "Water"
    set RBW "25.00"
}
```

Tech note: The 3 slices must show the center portion of lesion (e.g. largest cross section). Auto-prescan performed for this scan. This is a breathhold scan (approx 30 seconds). The scan can be changed to two 15 second breathholds by changing # acquisitions before pause. Follow the breathing instructions to the patient described in the general guidelines (*vide supra*). Note that TE 1.8 ms was available by choosing "out-of-phase" TE selection. Minimum TR is automatically chosen with this sequence. Using Bandwidth 25.0 kHz, the minimum TR was 6.4 ms. The next available higher bandwidth was 31.25 KHz, and the minimum TR was 5.9 ms. The next available lower bandwidth was 15.63 KHz, and the TR was 8.2 ms. Bandwidth of 25.0 KHz is used. Choose Bandwidth such that TR is as close as possible to 6.4 ms.

6. Repeat of scan 5 but with flip angle 15 degrees

```
    set PLANE "AXIAL"
    set SEDESC "FSPGR Pre FA15 10 acq 3-slice"
    set IMODE "2D"
    set PSEQ "SPGR"
    set IOPT "Seq, VBw, EDR, Fast, MPh"
    set SLPERLOC "10"
    set DELACQ "Minimum"
    set FLIPANG "15"
    set TE "Out of Phase" (sets TE to 1.8 ms)
    set NECHO "1"
    set FOV "32"
    set SLTHICK "8.0"
    set SPC "1.0"
    set NOSLC "3"
    set MATRIXX "256"
    set MATRIXY "160"
    set SWAPPF "R/L"
    set NEX "1.00"
    set PHASEFOV "1.00"
    set AUTOCF "Water"
    set RBW "25.00"
```

Tech note: It is critical to forming the T1 map that a prescan is NOT performed prior to this scan. To avoid prescan, select Manual Prescan, then select End on the Manual Prescan page (not Cancel). This is a breathhold

scan (approx 30 seconds). The scan can be changed to two 15 second breathholds by changing # acquisitions before pause. Follow the breathing instructions to the patient described in the general guidelines (*vide supra*).

7. Repeat of scan 5 but with flip angle 3 degrees

```
set PLANE "AXIAL"
set SEDESC "FSPGR Pre FA03 10 acq 3-slice"
set IMODE "2D"
set PSEQ "SPGR"
set IOPT "Seq, VBw, EDR, Fast, MPh"
set SLPERLOC "10"
set DELACQ "Minimum"
set FLIPANG "3"
set TE "Out of Phase" (sets TE to 1.8 ms)
set NECHO "1"
set FOV "32"
set SLTHICK "8.0"
set SPC "1.0"
set NOSLC "3"
set MATRIXX "256"
set MATRIXY "160"
set SWAPPF "R/L"
set NEX "1.00"
set PHASEFOV "1.00"
set AUTOCF "Water"
set RBW "25.00"
```

Tech note: It is critical to forming the T1 map that a prescan is NOT performed prior to this scan. To avoid prescan, select Manual Prescan, then select End on the Manual Prescan page (not Cancel). This is a breathhold scan (approx 30 seconds). The scan can be changed to two 15 second breathholds by changing # acquisitions before pause. Follow the breathing instructions to the patient described in the general guidelines (*vide supra*).

8. Repeat of scan 5 with flip angle 30 degrees but with 40 to 48 acquisitions (8 minutes), 3 slices

```
set PLANE "AXIAL"
set SEDESC "FSPGR INFUS FA30 160 acq 3-sl"
set IMODE "2D"
set PSEQ "SPGR"
set IOPT "Seq, VBw, EDR, Fast, MPh"
set SLPERLOC "30"
set DELACQ "Minimum"
set FLIPANG "30"
set TE "Out of Phase"
set NECHO "1"
set FOV "32"
set SLTHICK "8.0"
set SPC "1.0"
set NOSLC "1"
set MATRIXX "256"
set MATRIXY "160"
set SWAPPF "R/L"
set NEX "1.00"
set CONTRAST "Yes"
set PHASEFOV "1.00"
```

```
set AUTOCF "Water"
set RBW "25.00"
```

Tech note: It is critical to relating this data to the T1 map that a prescan is NOT performed prior to this scan. To avoid prescan, select Manual Prescan, then select End on the Manual Prescan page (not Cancel).

Tech note: The breathing instructions to the patient can be similar to those described in the general guidelines (vide supra). However, because imaging time is 1 second per slice, up to 7 slices can be acquired per breathhold period using the general guidelines. Alternatively, a 10-12 second temporal resolution of the dynamic contrast enhancement curve in 3 slice locations can be obtained using a 3 second acquisition, combined with a 7-9 second breathhold. For this acquisition, the instructions are as follows: 1. Instruct the patient to “breathe in, breathe out, breathe in, breathe out, and hold”, and acquire one set of three pre-Gd images during this first exhalation (HE). After this acquisition, instruct the patient to “breathe in, breathe out, hold”, and acquire another set of three images. After this acquisition, instruct the patient to “breathe in, breathe out, hold”, and acquire another set of three images. After this acquisition, start injection, and instruct the patient to “breathe in, breathe out, hold”, and acquire another set of three images. Continue instructing the patient to “Breathe in, Breathe out, Hold” and acquiring additional sets of three images. Each cycle requires 3 seconds of scan time plus time to breathe in and breathe out, or 10-12 seconds per cycle, depending on patient tolerance. This time per cycle establishes the temporal resolution of the DCE-MRI study, and should be maintained constant through the first four minutes of the study.

Repeat “Breathe in, Breathe out, Hold” instructions at a 10-12 second intervals for 4 minutes post-injection. Monitor the pulse oximeter to determine that the patient is tolerating the repeated breath-holding. When the patient cannot maintain breathhold during image acquisition, switch to a longer breathing instruction. In any case, at 4 min post-injection, switch to a longer breathing instruction. The longer instruction is “Breathe in, Breathe out, Breathe in, Breathe out, Hold” before each 3-slice acquisition. This longer instruction increases the time interval to around 20-24 seconds per cycle, depending on the patient. Continue image acquisition out to 8 min post-injection.

Tech note: Power injector must be used for contrast injection. Gd-based contrast agent (e.g., Omniscan) required. Use 0.1 mmoles/kg of agent (the standard product concentration) followed by 20 cc saline flush.

Tech note: Note that TE: 1.8 ms was available by choosing “out-of-phase” TE selection. Minimum TR is automatically chosen with this sequence. Using Bandwidth 25.0 kHz, the minimum TR was 6.4 ms. The next available higher bandwidth was 31.25 KHz, and the minimum TR was 5.9 ms. The next available lower bandwidth was 15.63 KHz, and the minimum TR was 8.2 ms. Bandwidth of 25.0 KHz is used. Choose Bandwidth such that TR is as close as possible to 6.4 ms.

9. Post-infusion T1 weighted scan (same parameters as Scan 4):

```
set PLANE "AXIAL"
set SEDESC "FSPGR POST TE 1.8 All-slice"
set IMODE "2D"
set PSEQ "SPGR"
set IOPT "VBw, EDR, Fast"
set FLIPANG "60"
set TE "Out of Phase"
set NECHO "1"
set TR "100.0"
set FOV "32"
set SLTHICK "8.0"
set SPC "1.0"
set NOSLC "18"
set MATRIXX "256"
set MATRIXY "160"
```

```
set SWAPPF "R/L"
set NEX "1.00"
set CONTRAST "Yes"
set PHASEFOV "1.00"
set AUTOCF "Water"
set RBW "15.63"
```

Tech notes: Be sure to use same slice locations as used in scan 3. Perform Automatic Prescan for this scan. However, copy the receiver gain setting from scan 3 (Series Textpage in Browser). This is post-contrast, free-breathing scan. This scan is companion to pre-infusion T1 weighted scan (Series 3).

GLOSSARY OF TERMS USED IN PROTOCOL DEFINITION:

```
PLANE: slice orientation
SEDESC: Text description of sequence
IMODE: Imaging Mode (e.g., 2D, 3D, CINE, spectro)
PSEQ: Pulse sequence type (e.g. Localizer, Spin Echo, Grad Echo)
PSDNAME: Pulse sequence description binary file name
IOPT: Imaging Options
      Fast: Minimum TR using fast gradient switching
      VBw: Variable receiver bandwidth
      EDR: Extended dynamic range for receiver
      MPh: Multi-phase acquisition (repeated volume acquisition)
      Seq: Sequential acquisition
SLPERLOC: Slices per location (repetitions of each slice)
DELACQ: time delay in ms after each completed volume acquisition
SATTHICKZ1: Superior SAT pulse thickness (using 30 mm gap)
SATTHICKZ2: Inferior SAT pulse thickness (using 30 mm gap)
FLIPANG: RF pulse flip angle in degrees (default is 90)
TE: Echo Time in ms
NECHO: number of echos in each TR
TR: Repetition time in ms
ETL: Echo train length for fast spin echo sequences
FOV: Field of View (both directions)
SLTHICK: Slice thickness in mm
SL3PLANE: Number of slices in each plane (3-plane PLANE only)
SPC: slice spacing in mm
NOSLC: Number of slices
MATRIXX: K-space data points in frequency encode (x) direction
MATRIXY: K-space data points in phase encode (y) direction
SWAPPF: Provides direction of frequency encoding (e.g R/L)
 "Unswap": standard frequency encoding direction
NEX: Number of excitations
PHASEFOV: Faction of full FOV for phase encode direction
AUTOCF: RF transmit and receiver frequency center
RBW: Receiver Bandwidth in KHz
AUTOSHIM: Yes or No for system to do Automatic shimming
```

14.1.3 Toronto Extremity Salvage Score (appended)

14.1.4 SF-36 Health Survey (appended)

15.0 REFERENCES

1. Jemal A, Siegel R, Ward E, et al. Cancer statistics, 2008. CA Cancer J Clin 2008; 58(2):71-96.
2. Lewis JJ, Brennan MF. Soft tissue sarcomas. Curr Probl Surg 1996; 33(10):817-72.
3. Kattan MW, Leung DH, Brennan MF. Postoperative nomogram for 12-year sarcoma-specific death. J Clin Oncol 2002; 20(3):791-6.
4. Wunder JS, Nielsen TO, Maki RG, et al. Opportunities for improving the therapeutic ratio for patients with sarcoma. Lancet Oncol 2007; 8(6):513-24.
5. Weitz J, Antonescu CR, Brennan MF. Localized extremity soft tissue sarcoma: improved knowledge with unchanged survival over time. J Clin Oncol 2003; 21(14):2719-25.
6. Yang JC, Chang AE, Baker AR, et al. Randomized prospective study of the benefit of adjuvant radiation therapy in the treatment of soft tissue sarcomas of the extremity. J Clin Oncol 1998; 16(1):197-203.
7. Pisters PW, Harrison LB, Leung DH, et al. Long-term results of a prospective randomized trial of adjuvant brachytherapy in soft tissue sarcoma. J Clin Oncol 1996; 14(3):859-68.
8. O'Sullivan B, Davis AM, Turcotte R, et al. Preoperative versus postoperative radiotherapy in soft-tissue sarcoma of the limbs: a randomised trial. Lancet 2002; 359(9325):2235-41.
9. Davis AM, O'Sullivan B, Turcotte R, et al. Late radiation morbidity following randomization to preoperative versus postoperative radiotherapy in extremity soft tissue sarcoma. Radiother Oncol 2005; 75(1):48-53.
10. Eilber FC, Brennan MF, Eilber FR, et al. Validation of the postoperative nomogram for 12-year sarcoma-specific mortality. Cancer 2004; 101(10):2270-5.
11. Mariani L, Miceli R, Kattan MW, et al. Validation and adaptation of a nomogram for predicting the survival of patients with extremity soft tissue sarcoma using a three-grade system. Cancer 2005; 103(2):402-8.
12. Adjuvant chemotherapy for localised resectable soft-tissue sarcoma of adults: meta-analysis of individual data. Sarcoma Meta-analysis Collaboration. Lancet 1997; 350(9092):1647-54.
13. Frustaci S, De Paoli A, Bidoli E, et al. Ifosfamide in the adjuvant therapy of soft tissue sarcomas. Oncology 2003; 65 Suppl 2:80-4.
14. Frustaci S, Gherlinzoni F, De Paoli A, et al. Adjuvant chemotherapy for adult soft tissue sarcomas of the extremities and girdles: results of the Italian randomized cooperative trial. J Clin Oncol 2001; 19(5):1238-47.
15. Gortzak E, Azzarelli A, Buesa J, et al. A randomised phase II study on neo-adjuvant chemotherapy for 'high-risk' adult soft-tissue sarcoma. Eur J Cancer 2001; 37(9):1096-103.
16. Kraybill WG, Harris J, Spiro IJ, et al. Phase II study of neoadjuvant chemotherapy and radiation therapy in the management of high-risk, high-grade, soft tissue sarcomas of the extremities and body wall: Radiation Therapy Oncology Group Trial 9514. J Clin Oncol 2006; 24(4):619-25.
17. Petrioli R, Coratti A, Correale P, et al. Adjuvant epirubicin with or without Ifosfamide for adult soft-tissue sarcoma. Am J Clin Oncol 2002; 25(5):468-73.

18. Pisters PW, O'Sullivan B, Maki RG. Evidence-based recommendations for local therapy for soft tissue sarcomas. J Clin Oncol 2007; 25(8):1003-8.
19. Jain RK. Tumor angiogenesis and accessibility: role of vascular endothelial growth factor. Semin Oncol 2002; 29(6 Suppl 16):3-9.
20. Jain RK. Antiangiogenic therapy for cancer: current and emerging concepts. Oncology (Williston Park) 2005; 19(4 Suppl 3):7-16.
21. Jain RK. Angiogenesis and lymphangiogenesis in tumors: insights from intravital microscopy. Cold Spring Harb Symp Quant Biol 2002; 67:239-48.
22. Hurwitz H, Fehrenbacher L, Novotny W, et al. Bevacizumab plus irinotecan, fluorouracil, and leucovorin for metastatic colorectal cancer. N Engl J Med 2004; 350(23):2335-42.
23. Miller K, Wang M, Gralow J, et al. Paclitaxel plus bevacizumab versus paclitaxel alone for metastatic breast cancer. N Engl J Med 2007; 357(26):2666-76.
24. Sandler A, Gray R, Perry MC, et al. Paclitaxel-carboplatin alone or with bevacizumab for non-small-cell lung cancer. N Engl J Med 2006; 355(24):2542-50.
25. Yoon SS, Segal NH, Olshen AB, et al. Circulating angiogenic factor levels correlate with extent of disease and risk of recurrence in patients with soft tissue sarcoma. Ann Oncol 2004; 15(8):1261-6.
26. Yoon SS, Segal NH, Park PJ, et al. Angiogenic profile of soft tissue sarcomas based on analysis of circulating factors and microarray gene expression. J Surg Res 2006; 135(2):282-90.
27. Chao C, Al-Saleem T, Brooks JJ, et al. Vascular endothelial growth factor and soft tissue sarcomas: tumor expression correlates with grade. Ann Surg Oncol 2001; 8(3):260-7.
28. Hayes AJ, Mostyn-Jones A, Koban MU, et al. Serum vascular endothelial growth factor as a tumour marker in soft tissue sarcoma. Br J Surg 2004; 91(2):242-7.
29. Graeven U, Andre N, Achilles E, et al. Serum levels of vascular endothelial growth factor and basic fibroblast growth factor in patients with soft-tissue sarcoma. J Cancer Res Clin Oncol 1999; 125(10):577-81.
30. Yudoh K, Kanamori M, Ohmori K, et al. Concentration of vascular endothelial growth factor in the tumour tissue as a prognostic factor of soft tissue sarcomas. Br J Cancer 2001; 84(12):1610-5.
31. D'Adamo DR, Anderson SE, Albritton K, et al. Phase II study of doxorubicin and bevacizumab for patients with metastatic soft-tissue sarcomas. J Clin Oncol 2005; 23(28):7135-42.
32. Li J, Huang S, Armstrong EA, et al. Angiogenesis and radiation response modulation after vascular endothelial growth factor receptor-2 (VEGFR2) blockade. Int J Radiat Oncol Biol Phys 2005; 62(5):1477-85.
33. Plastaras JP, Kim SH, Liu YY, et al. Cell cycle dependent and schedule-dependent antitumor effects of sorafenib combined with radiation. Cancer Res 2007; 67(19):9443-54.
34. Czito BG, Bendell JC, Willett CG, et al. Bevacizumab, oxaliplatin, and capecitabine with radiation therapy in rectal cancer: Phase I trial results. Int J Radiat Oncol Biol Phys 2007; 68(2):472-8.
35. Zhu AX, Willett CG. Chemotherapeutic and biologic agents as radiosensitizers in rectal cancer. Semin Radiat Oncol 2003; 13(4):454-68.

36. Senan S, Smit EF. Design of clinical trials of radiation combined with antiangiogenic therapy. Oncologist 2007; 12(4):465-77.
37. Benjamin RS, Choi H, Macapinlac HA, et al. We should desist using RECIST, at least in GIST. J Clin Oncol 2007; 25(13):1760-4.
38. Eilber FC, Rosen G, Eckardt J, et al. Treatment-induced pathologic necrosis: a predictor of local recurrence and survival in patients receiving neoadjuvant therapy for high-grade extremity soft tissue sarcomas. J Clin Oncol 2001; 19(13):3203-9.
39. Hew L, Kandel R, Davis A, et al. Histological necrosis in soft tissue sarcoma following preoperative irradiation. J Surg Oncol 1994; 57(2):111-4.
40. Willett CG, Schiller AL, Suit HD, et al. The histologic response of soft tissue sarcoma to radiation therapy. Cancer 1987; 60(7):1500-4.
41. Wilhelm SM, Carter C, Tang L, et al. BAY 43-9006 exhibits broad spectrum oral antitumor activity and targets the RAF/MEK/ERK pathway and receptor tyrosine kinases involved in tumor progression and angiogenesis. Cancer Res 2004; 64(19):7099-109.
42. Chang YS, Adnane J, Trail PA, et al. Sorafenib (BAY 43-9006) inhibits tumor growth and vascularization and induces tumor apoptosis and hypoxia in RCC xenograft models. Cancer Chemother Pharmacol 2007; 59(5):561-74.
43. Awada A, Hendlisz A, Gil T, et al. Phase I safety and pharmacokinetics of BAY 43-9006 administered for 21 days on/7 days off in patients with advanced, refractory solid tumours. Br J Cancer 2005; 92(10):1855-61.
44. Strumberg D, Richly H, Hilger RA, et al. Phase I clinical and pharmacokinetic study of the Novel Raf kinase and vascular endothelial growth factor receptor inhibitor BAY 43-9006 in patients with advanced refractory solid tumors. J Clin Oncol 2005; 23(5):965-72.
45. Escudier B, Eisen T, Stadler WM, et al. Sorafenib in advanced clear-cell renal-cell carcinoma. N Engl J Med 2007; 356(2):125-34.
46. Ratain MJ, Eisen T, Stadler WM, et al. Phase II placebo-controlled randomized discontinuation trial of sorafenib in patients with metastatic renal cell carcinoma. J Clin Oncol 2006; 24(16):2505-12.
47. Medved M, Karczmar G, Yang C, et al. Semiquantitative analysis of dynamic contrast enhanced MRI in cancer patients: Variability and changes in tumor tissue over time. J Magn Reson Imaging 2004; 20(1):122-8.
48. Tofts PS. Modeling tracer kinetics in dynamic Gd-DTPA MR imaging. J Magn Reson Imaging 1997; 7(1):91-101.
49. Padhani AR, Husband JE. Dynamic contrast-enhanced MRI studies in oncology with an emphasis on quantification, validation and human studies. Clin Radiol 2001; 56(8):607-20.
50. Kamel IR, Bluemke DA, Eng J, et al. The role of functional MR imaging in the assessment of tumor response after chemoembolization in patients with hepatocellular carcinoma. J Vasc Interv Radiol 2006; 17(3):505-12.
51. Kamel IR, Reyes DK, Liapi E, et al. Functional MR imaging assessment of tumor response after 90Y microsphere treatment in patients with unresectable hepatocellular carcinoma. J Vasc Interv Radiol 2007; 18(1 Pt 1):49-56.

52. Reddick WE, Wang S, Xiong X, et al. Dynamic magnetic resonance imaging of regional contrast access as an additional prognostic factor in pediatric osteosarcoma. Cancer 2001; 91(12):2230-7.
53. Dyke JP, Panicek DM, Healey JH, et al. Osteogenic and Ewing sarcomas: estimation of necrotic fraction during induction chemotherapy with dynamic contrast-enhanced MR imaging. Radiology 2003; 228(1):271-8.
54. Fletcher BD, Hanna SL, Fairclough DL, Gronemeyer SA. Pediatric musculoskeletal tumors: use of dynamic, contrast-enhanced MR imaging to monitor response to chemotherapy. Radiology 1992; 184(1):243-8.
55. Lim JH, Lee ES, You HJ, et al. Ras-dependent induction of HIF-1alpha785 via the Raf/MEK/ERK pathway: a novel mechanism of Ras-mediated tumor promotion. Oncogene 2004; 23(58):9427-31.
56. Lu Y, Liang K, Li X, Fan Z. Responses of cancer cells with wild-type or tyrosine kinase domain-mutated epidermal growth factor receptor (EGFR) to EGFR-targeted therapy are linked to downregulation of hypoxia-inducible factor-1alpha. Mol Cancer 2007; 6:63.
57. Therasse P, Arbuck SG, Eisenhauer EA, et al. New guidelines to evaluate the response to treatment in solid tumors. European Organization for Research and Treatment of Cancer, National Cancer Institute of the United States, National Cancer Institute of Canada. J Natl Cancer Inst 2000; 92(3):205-16.
58. Galbraith SM, Lodge MA, Taylor NJ, et al. Reproducibility of dynamic contrast-enhanced MRI in human muscle and tumours: comparison of quantitative and semi-quantitative analysis. NMR Biomed 2002; 15(2):132-42.
